# Concentration and Subclass Distribution of Anti-ADAMTS13 IgG Autoantibodies in Different Stages of Acquired Idiopathic Thrombotic Thrombocytopenic Purpura

**DOI:** 10.3389/fimmu.2018.01646

**Published:** 2018-07-16

**Authors:** György Sinkovits, Ágnes Szilágyi, Péter Farkas, Dóra Inotai, Anikó Szilvási, Attila Tordai, Katalin Rázsó, Marienn Réti, Zoltán Prohászka

**Affiliations:** ^1^Research Laboratory, 3rd Department of Internal Medicine and MTA-SE Research Group of Immunology and Hematology, Hungarian Academy of Sciences and Semmelweis University, Budapest, Hungary; ^2^3rd Department of Internal Medicine, Semmelweis University, Budapest, Hungary; ^3^Laboratory of Transplantation Immunogenetics, Hungarian National Blood Transfusion Service, Budapest, Hungary; ^4^Department of Pathophysiology, Semmelweis University, Budapest, Hungary; ^5^Division of Haematology, Deptartment of Internal Medicine, Faculty of Medicine, University of Debrecen, Debrecen, Hungary; ^6^Department of Haematology and Stem Cell Transplantation, Central Hospital of Southern Pest, National Institute of Haematology and Infectious Diseases, Budapest, Hungary

**Keywords:** thrombotic thrombocytopenic purpura, anti-ADAMTS13 autoantibodies, IgG subclasses, ADAMTS13-inhibitors, HLA-DRB1-DQB1 haplotypes

## Abstract

**Background:**

The acquired form of idiopathic thrombotic thrombocytopenic purpura (TTP) is an autoimmune disease, in which the underlying deficiency of the ADAMTS13 protease is caused by autoantibodies, predominantly of the IgG isotype. Certain HLA-DR-DQ haplotypes were associated with the risk of developing TTP.

**Objectives:**

To investigate the development of the ADAMTS13-specific antibody response during the course of the disease, we analyzed the concentration, subclass distribution, and inhibitory potential of anti-ADAMTS13 IgG autoantibodies in samples of TTP patients drawn during the first acute phase, in remission, and during relapse. Additionally, we compared the anti-ADAMTS13 IgG levels between patients carrying and not carrying risk and protective HLA-DR-DQ haplotypes.

**Patients and Methods:**

We determined the anti-ADAMTS13 IgG concentration and subclass distribution in 101 antibody-positive samples of 81 acquired TTP patients by ELISA methods. The presence and semi-quantitative amount of anti-ADAMTS13 inhibitors were determined in 97 of 100 deficient samples, and the specific inhibitory potential of anti-ADAMTS13 autoantibodies was determined in 49 selected samples, by mixing ADAMTS13-activity assays. HLA-DR-DQ typing and haplotype prediction were performed in 70 of the above patients.

**Results:**

We found that IgG1 and IgG4 were the predominant subclasses, present in almost all samples. While IgG1 was the dominant subclass in almost half of the samples taken during the first acute episode, IgG4 was dominant in all samples taken during or following a relapse. The inhibitory potential of the samples correlated with levels of the IgG4 subclass. Anti-ADAMTS13 antibodies of IgG4-dominant samples had higher specific inhibitory potentials than IgG1-dominant samples, independently of disease stage. Interestingly, we found that patients carrying the protective DR7-DQ2 and DR13-DQ6 haplotypes had higher anti-ADAMTS13 IgG levels.

**Conclusion:**

Our results indicate that IgG4 becomes the dominant subtype at some point of the disease course, apparently before the first relapse, parallel to the increase in inhibitory potential of the anti-ADAMTS13 autoantibodies. Furthermore, we found an association between the genetic background and the antibody response in TTP.

## Introduction

Idiopathic thrombotic thrombocytopenic purpura (TTP) is a rare but life-threatening disease, which belongs to the group of thrombotic microangiopathies, and presents with episodes of severe thrombocytopenia, microangiopathic hemolytic anemia (MAHA) with fragmentation of erythrocytes, and end-organ dysfunction caused by microvascular thrombosis (1). The pathogenic thrombi consist primarily of platelets bound to the ultra-large form of Von Willebrand factor (ULVWF) (2, 3). ULVWF multimers are supposed to be cleaved by the ADAMTS13 protease (4, 5). In the idiopathic form of TTP, however, the activity of the ADAMTS13 enzyme is deficient, resulting in increased levels of ULVWF, which are able to bind and activate platelets (2, 6).

The underlying ADAMTS13 deficiency is caused by *ADAMTS13* mutations in the rare, congenital form of TTP (7), whereas the more common, acquired form of TTP is an autoimmune disease, in which autoantibodies against the ADAMTS13 enzyme are responsible for its deficiency (8, 9).

Some of these antibodies are inhibitory, directly blocking the enzymatic activity of the protease (8, 9), while some are non-inhibitory (10). Irrespective of the inhibitory potential of the autoantibodies, they can also contribute to the ADAMTS13 deficiency by promoting the clearance of the enzyme from the circulation (11–13).

Anti-ADAMTS13 autoantibodies are predominantly of the IgG isotype, although IgM and IgA class antibodies have also been described in some cases (10, 14–18). IgG antibodies can be subdivided into four subclasses based on differences in their Fc regions. These differences affect their ability to bind complement or Fc receptors of effector cells, resulting in distinct immunological properties. Most anti-ADAMTS13 antibodies belong to the IgG1 and IgG4 subclasses (16–19); IgG1 and IgG3 levels were found to be associated with the clinical severity of the episode (16, 17) and IgG4 levels with the risk of relapse (16).

Relapses (acute episodes following complete remission) occur in about one-third of TTP patients (20). Anti-ADAMTS13 autoantibody levels are usually higher during the acute episodes, and lower or undetectable during remission. However, free antibodies or immune complexes may also be present during remission, leading to deficient ADAMTS13-activity in a subset of remission patients (18, 21), which increases the risk of disease relapse (15, 21).

During the disease course often spanning over decades, the immune response against ADAMTS13 may go through certain changes in response to the prolonged antigen stimulation or to the various therapies. The primary goal of this study was to investigate the changes in the immune response by comparing immunological properties (concentration, subclass distribution, and inhibitory potential) of the anti-ADAMTS13 IgG autoantibodies in different disease stages.

The antibody response against a protein antigen and isotype switching of autoantibodies to the IgG class implies the role of helper T cells and antigen presentation in the development of acquired TTP. During antigen presentation, peptides of extracellular protein antigens are presented to CD4-positive helper T cells *via* the HLA-DR and DQ antigens, which are inherited in linkage in the form of DR-DQ haplotypes. Indeed, it has been shown that certain HLA-DR and DQ alleles, or DR-DQ haplotypes are associated with increased or decreased risk of TTP (22–25). However, whether these HLA-DR and DQ alleles also influence the character of the immune response to ADAMTS13 has not yet been studied. Therefore, another aim of this study was to investigate whether the concentration and subclass distribution of anti-ADAMTS13 IgG antibodies differ in individuals carrying risk or protective HLA-DR-DQ haplotypes.

## Patients and Methods

### Patient and Sample Selection

We analyzed 101 anti-ADAMTS13 IgG-positive samples of 81 acquired TTP patients investigated in our laboratory between August 2007 and March 2017. Our laboratory is providing diagnostic services for patients from Hungary and nearby countries suspected of having thrombotic microangiopathies. The diagnosis of acquired TTP was based on the following criteria: (1) thrombocytopenia (platelet count below 150 G/L) and MAHA (Coombs-negative anemia, elevated LDH, schistocytes on the blood smear); (2) deficient ADAMTS13 activity [<10%, measured by the FRETS-VWF73 assay (fluorescence resonance energy transfer-based method using a 73-amino-acid synthetic peptide), as described below]; and (3) detectable anti-ADAMTS13 autoantibodies (by mixing activity assays or ELISA, as described below). One hundred and thirteen patients fulfilled the above criteria at a time point during the inclusion period.

Blood samples sent to our laboratory were taken either during an acute episode [before the initiation or during plasma exchange (PEX) therapy] or in remission. Complete remission was defined as normal platelet count (>150 G/L) and no signs of hemolysis, without new or worsening clinical symptoms for at least 30 days after stopping PEX therapy. (In the following, we refer to complete remission as ‘remission’.) Relapse was defined as a new TTP episode following remission.

The blood samples were obtained by venipuncture or from a central venous catheter; serum/plasma and cells were separated by centrifugation either in our laboratory, if transport time was less than 4 h, or on the site of blood collection. Samples were shipped to our laboratory in cooled packages (4°C) in case of whole blood samples, or frozen (−20°C) in case of separated samples, and were stored at −70°C until measurements. Sodium citrate-anticoagulated plasma or native serum samples were used for the determinations of ADAMTS13 activity and autoantibody concentrations.

**Figure 1:**
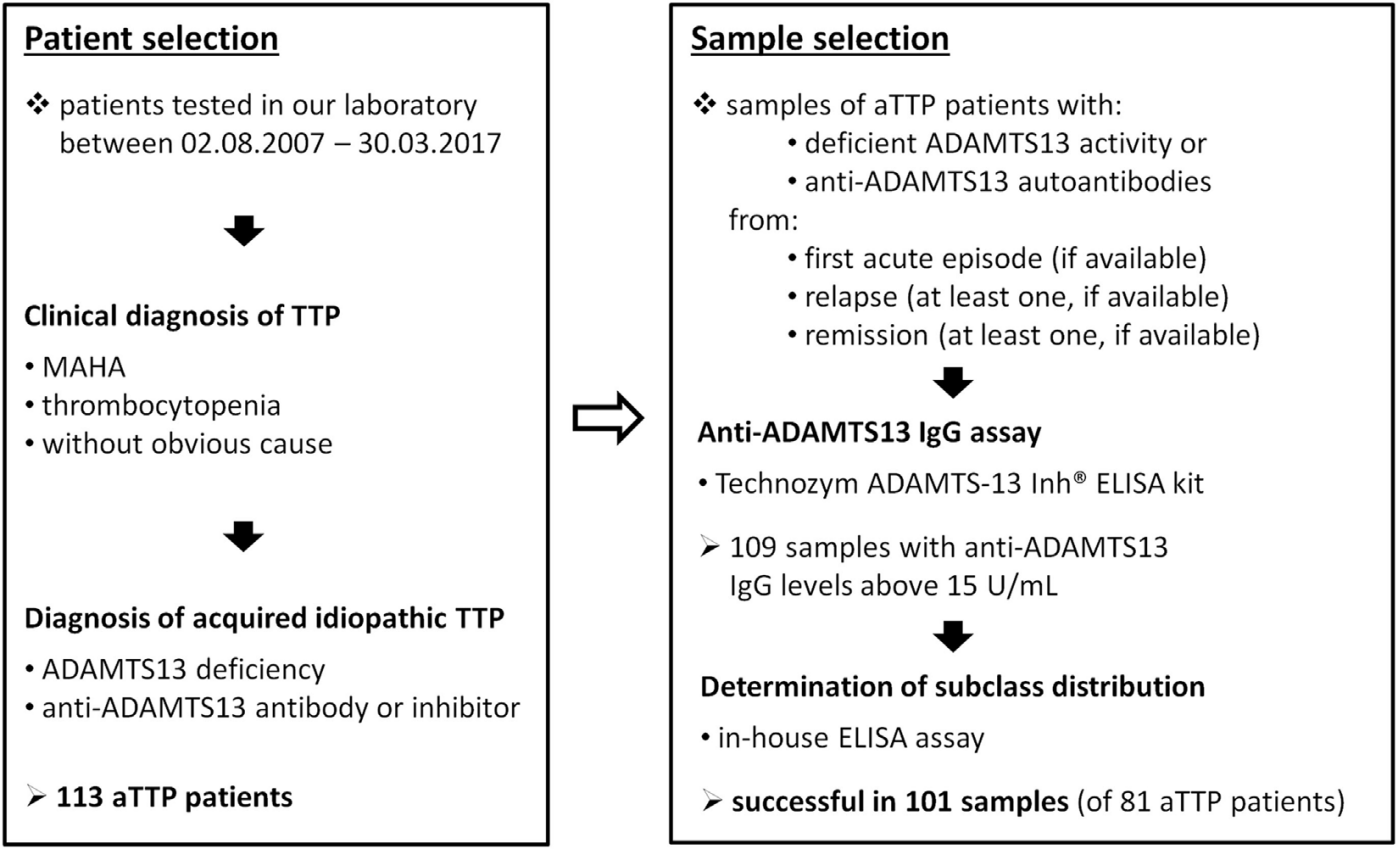
Scheme of patient and sample selection. Abbreviations: MAHA, microangiopathic hemolytic anemia; aTTP, acquired idiopathic thrombotic thrombocytopenic purpura.

All ADAMTS13-deficient (<10% activity) samples of acquired TTP patients available in our laboratory were tested for the presence of anti-ADAMTS13 IgG. A randomly selected subset of TTP patients’ samples with decreased but not deficient ADAMTS13 activities (between 20 and 40%) were also tested, but none of the 15 samples were anti-ADAMTS13 IgG positive. One hundred and nine available samples were found to be anti-ADAMTS13 IgG positive according to the Technozym ADAMTS13-Inh^®^ assay described below. These samples were selected for the subclass distribution analysis of anti-ADAMTS13 IgG autoantibodies. The determination was successful in 101 samples of 81 patients, 8 of the above samples contained too low antibody concentrations for the analysis. The steps of the patient and sample selection are summarized in Figure 1.

All available acute samples (from the first acute episode or from a relapse) with relative proportions of IgG4 over 70% or below 30%, and all available remission samples were selected for the determination of the specific inhibitory potentials of anti-ADAMTS13 autoantibodies. Thirty-five of the 50 selected acute samples and 14 of the 21 remission samples were available for the analysis in sufficient quantities.

Seventy of the included patients had available samples for DNA isolation, and HLA-DR and DQ typing.

The present study was conducted in accordance with the Declaration of Helsinki, written informed consent was obtained from all participating patients, and the study was approved by the Human Research Ethics Committee.

### Determination of ADAMTS13 Activity and Anti-ADAMTS13 Inhibitors

ADAMTS13 activity was measured by a FRETS-VWF73 assay described earlier (26) (AnaSpec Inc., Fremont, CA, USA).

The presence and semi-quantitative amount of functional anti-ADAMTS13 inhibitors were determined by measuring the ADAMTS13 activity of a 1:1 mixture of 50 µL deficient patient sample and 50 µL normal, pooled human citrated plasma or serum sample (with a nominal ADAMTS13 activity of 100%) following 2 h of incubation at 37°C. The activity of the mixed sample was compared to that of 100 µL incubated normal human sample (100%). An ADAMTS13 activity below 50% was considered positive for the presence of inhibitors, with lower activities implying stronger inhibition.

### Determination of the Total Anti-ADAMTS13 IgG Concentration

Total anti-ADAMTS13 IgG concentrations were measured by a commercial ELISA kit (Technozym ADAMTS-13 Inh^®^, Technoclone GmbH, Vienna, Austria). According to the manufacturer’s recommendation, samples with anti-ADAMTS13 IgG concentrations above 15 U/mL were considered positive.

### Determination of the Subclass Distribution of Anti-ADAMTS13 IgG Antibodies

We developed a novel ELISA-based method to precisely determine the subclass distribution of anti-ADAMTS13 IgG antibodies, which is described in detail in the Data Sheet S1 in Supplementary Material.

Briefly, we prepared calibrator plates by coating two columns of 96-well polystyrene microtiter plates (Greiner Bio One International GmbH, Kremsmünster, Austria) with known concentrations of purified human antibodies of each IgG subclass (Abcam, Cambridge, UK; Ref. No: IgG1—ab90283, IgG2—ab90284, IgG3—ab118426, IgG4—ab90286). We prepared twofold dilution series from each IgG subclass in bicarbonate buffer (pH 9.8), with concentrations ranging from 1,000 to 15.6 ng/mL and 0 ng/mL; 100 µL/well of these calibrators were loaded on the plate in duplicates and were incubated overnight at 4°C. The calibrator plates were washed, blocked by adding 110 µL/well of phosphate buffered saline (pH 7.4) containing 0.5% gelatine, and following an incubation of 50 min at room temperature, washed again three times by adding 200 µL/well of the washing buffer from the Technozym ADAMTS-13 Inh^®^ kit.

The samples were diluted in the diluent of the Technozym ADAMTS-13 Inh^®^ kit. The dilution factors were adjusted to the total anti-ADAMTS13 IgG concentrations of the samples, as described in the Data Sheet S1 in Supplementary Material; dilutions between 1:12.5 and 1:600 were used. 100 μL/well of each diluted sample was added to four wells of the test plate from the Technozym ADAMTS-13 Inh^®^ kit, coated with recombinant ADAMTS13 protein, and were incubated for 1 h at room temperature, simultaneously with the blocking of the calibrator plate. The plates were then washed five times.

Then, 100 μL/well of subclass-specific antibodies was added to the respective columns of the calibration plate and to one of the four wells containing a given sample on the test plate. We used HRP-conjugated mouse anti-human antibodies against the Fc region (Southern Biotech, Birmingham, AL, USA; Ref. No.: anti-IgG1—9054-05, anti-IgG2—9060-05, anti-IgG3—9210-05, anti-IgG4—9200-05) in the following dilutions: IgG1 and IgG2—1:1,000, IgG3—1:10,000, IgG4—1:5,000. Both (test and calibrator) plates were incubated for 1 h at room temperature, and washed six times. The plates were then developed by adding 100 μL/well of TMB (Novex^®^ TMB by life technologies, Frederick, MD, USA; Ref. No.: 00-2023). The substrate reaction was stopped by adding 50 μL/well of H_2_SO_4_ (4N) when the color reaction seemed to be optimal. Importantly, wells incubated by the same subclass-specific secondary antibodies were developed for the same time.

Absorbance of samples and calibrators was measured at 450 nm, with a reference wavelength of 620 nm. Calibrator curves were generated based on the optical density values of calibrator wells with known amount of bound antibodies for each subclass. The amounts of bound anti-ADAMTS13 antibodies of each IgG subclass were interpolated directly from the respective calibrator curve in the case of patient samples. Then, we determined the total amount of bound anti-ADAMTS13 antibodies by summing the amounts of all four subclasses. The proportion of each antibody subclass was calculated by dividing the bound amount of the respective subclass by the total amount of bound anti-ADAMTS13 antibodies.

### Calculation of the Absolute Concentrations of Anti-ADAMTS13 Antibodies of Distinct Subclasses

Absolute concentrations of anti-ADAMTS13 antibodies of each subclass were calculated by multiplying the total anti-ADAMTS13 IgG concentration (determined by the Technozym ADAMTS-13 Inh^®^ kit) by the proportion of the given subclass, determined as described above.

### Determination of the Specific Inhibitory Potential of Anti-ADAMTS13 Antibodies

Selected patient samples were diluted in phosphate buffered saline (pH 7.4) to a degree calculated by the total anti-ADAMTS13 IgG concentrations, in order to obtain equal final anti-ADAMTS13 IgG concentrations of 25 U/mL. The diluted samples were incubated with normal, pooled human citrated plasma or serum samples (according to the patient sample type; with nominal ADAMTS13 activity of 100%) in ratios of 3:1, 1:1, and 1:2 in a final volume of 60 µL, and were incubated for 2 h at 37°C. The ADAMTS13 activities of the mixed samples were measured as described above and compared to those of incubated pooled normal human samples (100%). A low ADAMTS13 activity of the mixed sample indicated a high specific inhibitory potential of the given anti-ADAMTS13 IgG antibodies and *vice versa*.

### HLA Typing and Haplotype Prediction

Low-resolution HLA-DRB1 and DQB1 typing was performed by polymerase chain reaction with sequence-specific oligonucleotide probe hybridization (PCR-SSO) (One Lambda, Canoga Park, CA, USA) at the Laboratory of Transplantation Immunogenetics in Budapest, and the HLA-DRB1-DQB1 haplotypes were predicted by the PHASE (v2.1) program (27, 28), as described previously (25).

### Statistical Analysis

As the distribution of variables was not normal in most cases, medians and interquartile (IQ) ranges are represented, and nonparametric statistical tests were used. Correlations between antibody concentrations and the ADAMTS13 activity of mixed samples were calculated by the Spearman rank correlation test. Antibody concentrations or proportions in distinct groups of patients were compared by the Mann–Whitney test. (When comparing parameters between two disease stages, the patients with paired samples in both disease stages were excluded in order that the two groups become independent.) The numbers of IgG1- or IgG4-dominant patients in two groups were compared by Fisher’s exact test. Two-way ANOVA was used to compare the ADAMTS13 activities of diluted patient samples mixed with pooled normal human samples. Findings with *p*-values below 0.05 were considered statistically significant. Statistical calculations were performed by the GraphPad Prism 5 software (GraphPad Softwares Inc., La Jolla, CA, USA).

## Results

### Description of the Patient Cohort

From the 113 patients in our registry who fulfilled the criteria of acquired idiopathic TTP during the inclusion period, 81 patients had anti-ADAMTS13 IgG-positive samples appropriate for the determination of IgG subclasses. Their median age was 38 (IQ range: 29–50) years; 62 (76.5%) of them were females and 19 (23.5%) were males. The median follow-up time was 21.9 (0.6–75.7) months, during which 26 patients (32.0%) had at least one relapse. Nine of 61 patients (14.8%) died during the first acute episode, and 1 of 19 patients (5.3%) died during the relapse in which the tested samples were drawn. Sixty-three acute samples were taken before the first PEX therapy, and 17 acute samples were taken following the first PEX session. Three relapse samples and eight remission samples were taken after rituximab therapy (one and six samples, respectively, were taken within 1 year of receiving rituximab). Other immunosuppressive drugs (cyclophosphamide, azathioprine, vincristine) were applied before the collection of four samples (two, two, and one, respectively).

### Anti-ADAMTS13 IgG Levels and Subclass Distribution in All Analyzed Acquired TTP Samples

**Figure 2:**
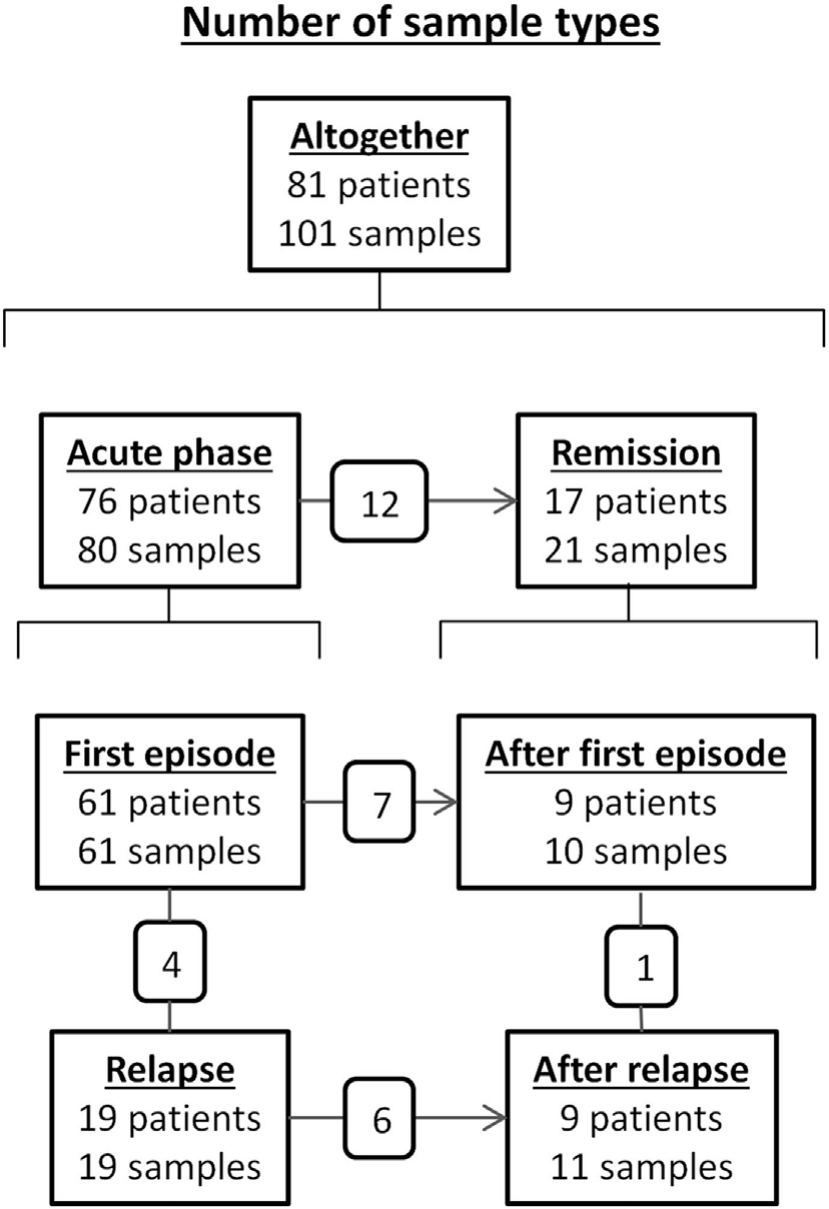
Number of patients and samples tested for anti-ADAMTS13 IgG subclass distribution according to disease stage. The number of common patients between disease stage groups are shown in the small boxes over the connecting lines.

We determined the concentration and subclass distribution of anti-ADAMTS13 IgG autoantibodies in 101 samples of the above described patients. The distribution of samples based on disease stage is summarized in Figure 2.

All tested samples were anti-ADAMTS13 IgG positive (above 15 U/mL). Median anti-ADAMTS13 IgG level was 66 U/mL, with an IQ range of 35.6–158.0 U/mL.

Anti-ADAMTS13 IgG antibodies were mostly of the IgG1 and IgG4 subclasses. IgG1 was the dominant subclass (i.e., the subclass with the highest proportion) in 28 of the 101 samples (27.7%), whereas IgG4 was dominant in 71 (70.3%) of them. IgG3 was the dominant subclass only in two samples (2.0%), whereas IgG2 was not dominant in any of the samples. The median proportion of IgG4 was the highest, 66.0% (31.5–76.0%) and that of IgG1 was 32.0% (24.0–51.5%), followed by IgG3 and IgG2 with 0.0% (0.0–5.5% and 0.0–2.5%, respectively). IgG1 was present in 97 samples (96.0%), whereas IgG4 was detectable in 94 of them (93.1%). IgG3 was detectable in 51 samples (50.5%), mostly in IgG1-dominant ones: it was present in 25 (89.3%) of the 28 IgG1-dominant samples. IgG2 could be detected in 41 samples (40.6%).

The proportion of neither of the IgG subclasses correlated with the total anti-ADAMTS13 IgG concentration. The concentrations of IgG subclasses correlated positively with each other, except for the concentrations of IgG3 and IgG4, which showed a strong negative correlation (*r* = −0.480, *p* < 0.0001).

There was no statistical difference between the total anti-ADAMTS13 IgG concentration and subclass distribution of acute samples taken before or after the first PEX therapy. The number of patients receiving rituximab or other immunosuppressive drugs was too low to perform statistical analyses, but their anti-ADAMTS13 IgG results were not substantially different from those of untreated patients.

### Anti-ADAMTS13 IgG Levels and Subclass Profile in Different Stages of the Disease

**Figure 3:**
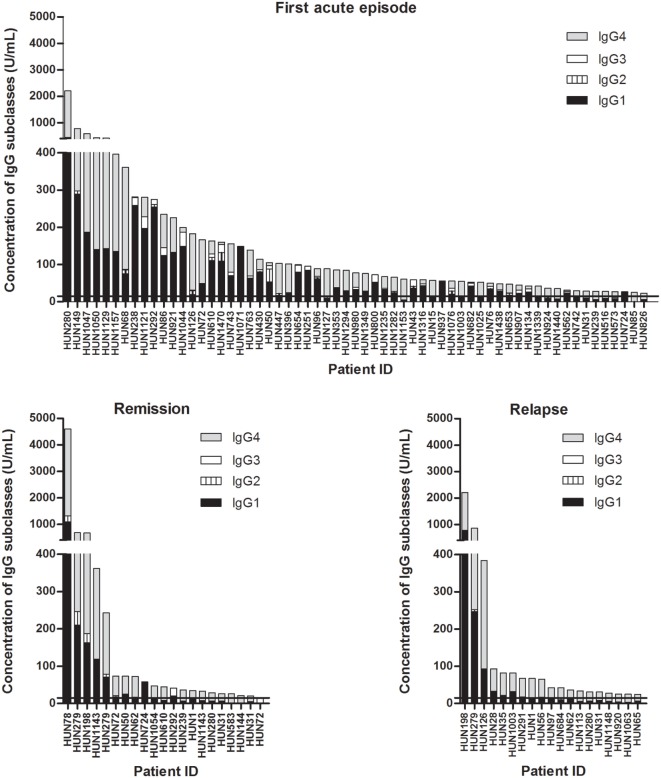
Concentration and subclass distribution of anti-ADAMTS13 IgG autoantibodies in the first acute phase, in relapse, and in remission. The horizontal line marks the cutoff value (15 U/mL).

Anti-ADAMTS13 IgG subclass concentrations in distinct disease stages are shown in Figure 3.

The anti-ADAMTS13 IgG concentrations and subclass distribution in independent samples drawn during the first acute phase and relapse are shown in Table 1. Patients with samples from both disease stages were excluded from the analysis. The age and gender distribution did not differ between the two groups. The total IgG levels tended to be higher in samples drawn in the first episode than in relapse [77.5 (47.6–161.8) vs. 42.3 (28.0–81.9) U/mL], but the difference was not statistically significant. During the first acute episode, IgG4 was the dominant subclass in 30 (52.6%) of the 57 samples, whereas IgG1 was dominant in 27 (47.4%) of them. In relapse samples, however, IgG4 was the dominant subclass in all 15 samples.

**Table 1:** Comparison of levels and subclass distribution of anti-ADAMTS13 IgG antibodies between the first acute episode and relapse.

	First episode	Relapse	
**Clinical data**			
Number of individuals	57	15	
Age (years)	41 (29–54)	35 (28–44)	
Gender (female/male)	42/15	12/3	
Current episode No	1	3 (2–5)	
Months since diagnosis of thrombotic thrombocytopenic purpura	0	69 (46–230)	
**Total anti-ADAMTS13 IgG concentration (U/mL)**			
IgG	77.5 (47.6–161.8)	42.3 (28.0–81.9)	*p* = 0.0831
**Proportion of IgG subclasses (%)**			
IgG1	**42.0 (30.6–68.8)**	**24.3 (21.0–28.8)**	***p* = 0.0002**
IgG2	0.0 (0.0–2.6)	1.1 (0.0–2.8)	*p* = 0.2230
IgG3	**2.2 (0.0–9.7)**	**0.0 (0.0–0.2)**	***p* = 0.0086**
IgG4	**49.7 (14.0–68.3)**	**73.6 (65.0–78.4)**	***p* = 0.0004**
**Concentration of IgG subclasses (U/mL)**			
IgG1	**35.7 (17.5–96.6)**	**12.8 (6.0–19.9)**	***p* = 0.0066**
IgG2	0.0 (0.0–2.8)	1.2 (0.0–3.8)	*p* = 0.1826
IgG3	**1.6 (0.0–8.2)**	**0.0 (0.0–0.1)**	***p* = 0.0061**
IgG4	25.2 (8.6–62.6)	28.5 (21.7–59.7)	*p* = 0.1552
**Dominant subclasses (number of patients, %)**			
IgG1	27 (47.4%)	0 (0%)	***p* = 0.0005**
IgG4	30 (52.6%)	15 (100%)	

*Results of independent samples are shown; patients having samples in both disease stages were excluded from the analysis in order to allow statistic comparison of the independent groups. (Similar results are obtained if all results are involved.) Values are expressed as median (interquartile range). The subclass with the highest proportion was considered dominant. Significantly different values are shown in bold*.

Similarly, IgG4-dominance could be observed in all 11 remission samples drawn after a relapse. In contrast, one sample drawn in remission following the first acute episode was IgG1-dominant and two samples were IgG3-dominant. The anti-ADAMTS13 IgG profiles in independent samples from remission following the first acute episode and following a relapse are shown in Table 2.

**Table 2:** Levels and subclass distribution of anti-ADAMTS13 IgG antibodies in clinical remission following the first acute episode and following a relapse.

	Remission after first episode	Remission after relapse
**Clinical data**		
Number of individuals	9	8
Age (years)	34 (20–45)	34 (30–45)
Gender (female/male)	8/1	8/0
No of previous episode	1	3 (2–4)
Months since first episode	7 (3–35)	72 (42–143)
**Total anti-ADAMTS13 IgG concentration (U/mL)**		
IgG	45.0 (31.5–73.3)	60.2 (30.6–592.2)
**Proportion of IgG subclasses (%)**		
IgG1	23.8 (19.2–41.7)	25.3 (22.1–31.5)
IgG2	0.0 (0.0–4.9)	0.0 (0.0–2.9)
IgG3	0.0 (0.0–2.0)	0.0 (0.0–0.6)
IgG4	71.4 (32.6–80.8)	73.0 (67.2–76.9)
**Concentration of IgG subclasses (U/mL)**		
IgG1	17.6 (6.7–39.4)	14.0 (8.1–152.0)
IgG2	0.0 (0.0–3.3)	0.0 (0.0–18.3)
IgG3	0.0 (0.0–1.0)	0.0 (0.0–0.3)
IgG4	29.6 (10.0–49.85)	45.7 (24.2–388.5)
**Dominant subclasses (number of patients)**		
IgG1 or IgG3	2 (22%)	0 (0%)
IgG4	7 (78%)	8 (100%)

*Results of independent samples are shown; only one (the first) remission sample of each patient was involved in the analysis. Values are expressed as median (IQ range). The subclass with the highest proportion was considered dominant. No statistical analysis was performed because of the low number of samples*.

Four patients had events of unexplained thrombocytopenia in their anamnesis prior to the diagnosis of TTP. Interestingly, all four of these patients’ samples, drawn during the episode when TTP was diagnosed, were IgG4 dominant.

**Figure 4:**
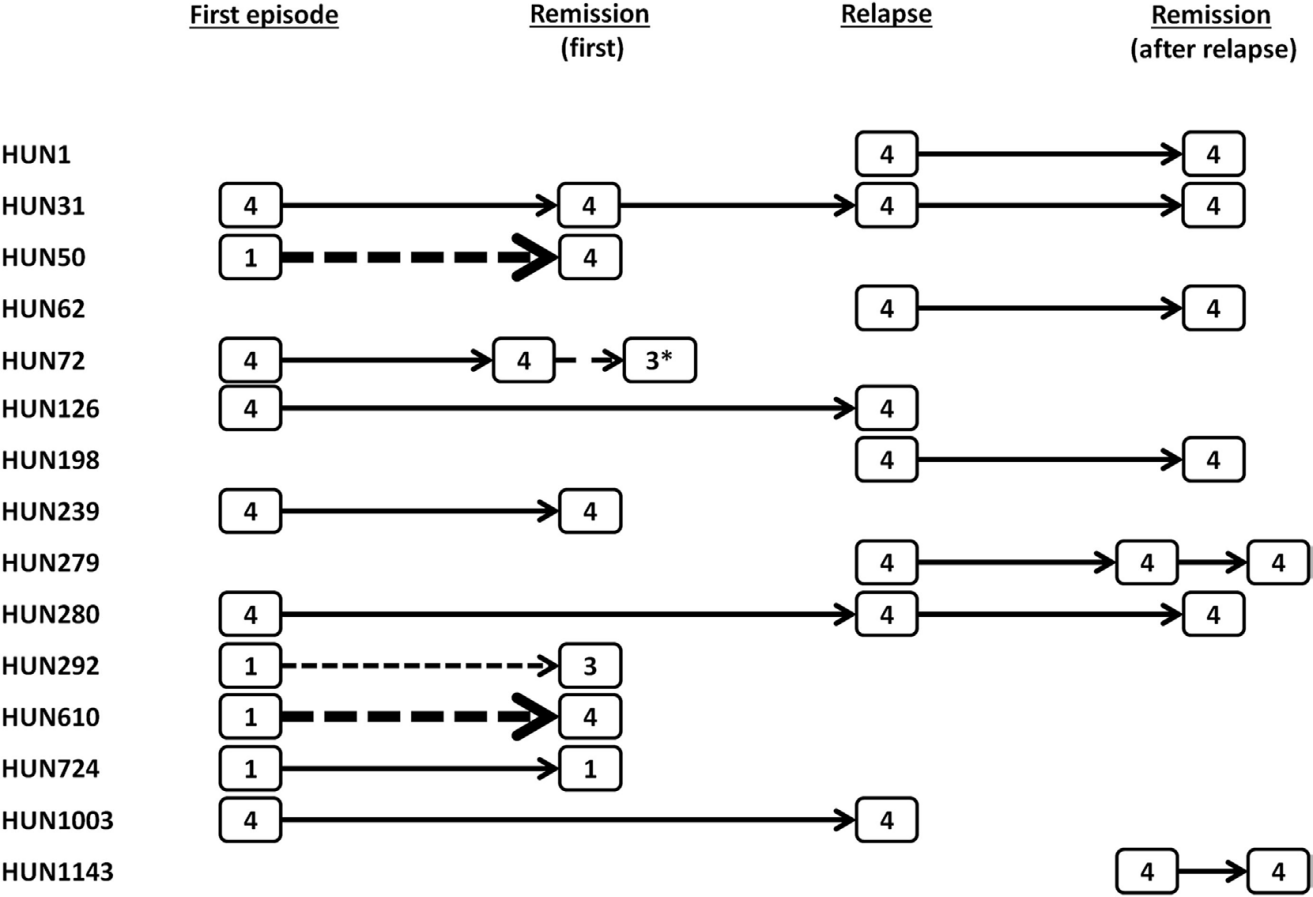
Dominant subclasses in samples of patients with multiple samples. Dashed arrows show a change in the dominant subclass, arrows indicating a change from IgG1 to IgG4 appear in bold. The only sample with normal ADAMTS13 activity (with low positive levels of anti-ADAMTS13 antibodies) is marked by an asterisk.

Fifteen patients had multiple samples, and the dominant subclasses in these samples are shown in Figure 4. The anti-ADAMTS13 IgG profiles of four patients with a sample from the first episode were initially IgG1-dominant, while all other patients were initially IgG4 dominant. In remission following the first acute episode, two of the four initially IgG1-dominant patients switched to an IgG4-dominant profile. However, in spite of the higher numbers of initially IgG4-dominant patients, a dominance switch in the other direction (from IgG4 to IgG1) was not observed. One initially IgG4-dominant patient had a non-deficient remission sample, though, which proved to be IgG3-dominant. All deficient samples taken during or following a relapse were IgG4 dominant.

Taken together, whereas a number of samples drawn during or following the first acute episode were IgG1- or IgG3-dominant, all deficient samples taken during or following a relapse were IgG4 dominant.

### ADAMTS13 Inhibition and Its Association with Anti-ADAMTS13 IgG Levels

ADAMTS13 activity was deficient in all samples except a remission sample with a low level (15.8 U/mL) of anti-ADAMTS13 IgG antibodies. The presence of anti-ADAMTS13 inhibitors was determined in 97 of the 100 deficient samples by a mixing FRET-vWF73 assay. The ADAMTS13 activity of patient samples mixed and incubated together with equal amounts of normal human plasma or serum [2.0% (0.0–11.5%)] implies the strength of the anti-ADAMTS13 inhibition in the sample, with lower activities indicating stronger inhibition and *vice versa*.

**Figure 5:**
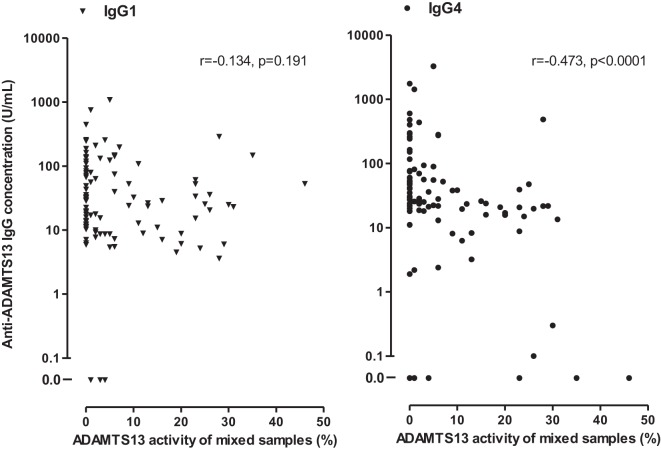
Associations of anti-ADAMTS13 IgG1 and IgG4 levels with the ADAMTS13 activity measured in 97 of the 100 deficient patient samples following mixing and incubation with equal amounts (1:1 ratio) of pooled normal human plasma or serum.

The ADAMTS13 activities of patient samples mixed and incubated together with equal amounts of pooled normal human samples showed a negative correlation with the total anti-ADAMTS13 IgG levels (*r* = −0.326, *p* = 0.011). However, an even stronger negative correlation was found with the levels of antibodies of the IgG4 subtype (*r* = −0.473, *p* < 0.0001) (shown in Figure 5), whereas no other subclass showed a correlation with the activities of mixed samples (IgG1: *r* = −0.134, *p* = 0.191; IgG2: *r* = −0.0127, *p* = 0.901; IgG3: *r* = 0.169, *p* = 0.098). The negative correlation between the activity of mixed samples and anti-ADAMTS13 IgG (*r* = −0.278, *p* = 0.034) or IgG4 (*r* = −0.302, *p* = 0.021) was also significant if only samples from the first acute phase were analyzed. Thus, the strength of ADAMTS13 inhibition, inferred from the activity of the mixed patient sample, correlated positively with anti-ADAMTS13 IgG4 levels.

### Specific Inhibitory Potential of Anti-ADAMTS13 Antibodies According to IgG Subclass Distribution and Disease Stage

**Figure 6:**
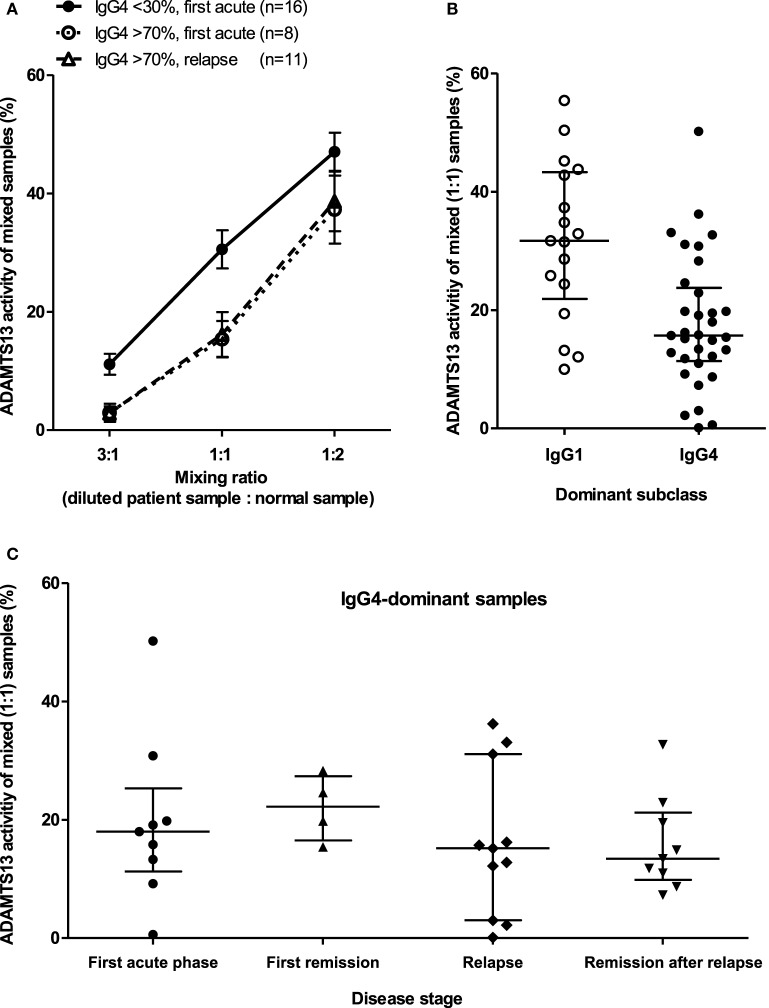
Specific inhibitory potentials of samples from different disease stages and with different IgG4 proportions. Patient samples were diluted in order to have equal (25 U/mL) anti-ADAMTS13 concentrations, diluted samples were mixed and incubated in different ratios with normal pooled human citrated plasma or serum, and ADAMTS13 activities of the mixed samples were measured. Low activities of mixed samples indicate a strong inhibitory potential and *vice versa*. **(A)** Specific inhibitory potentials of acute samples. Mean and SEM are shown for all mixing ratios. All deficient samples from the relapse phase were IgG4 dominant; none had IgG4 proportions below 30%. **(B)** Specific inhibitory potentials of all samples stratified by dominant IgG subclass. **(C)** Specific inhibitory potentials of IgG4 dominant samples stratified by disease stage. All deficient samples taken during or following a relapse were IgG4 dominant.

To directly assess the specific inhibitory potentials of patient samples, we diluted a selected set of samples to obtain equal (25 U/mL) anti-ADAMTS13 IgG concentrations. Thirty-five acute samples with low (<30%) or high (>70%) relative proportions of IgG4, and 14 remission samples were analyzed. The diluted samples were mixed and incubated with pooled normal human samples in three different ratios (3:1, 1:1, 1:2). The ADAMTS13 activities of the mixtures were compared according to disease stage and dominant IgG subclass, with lower activities indicating higher inhibitory potentials and *vice versa*. As it can be seen on Figure 6, IgG4-dominant samples clearly have a stronger inhibitory potential than IgG1-dominant samples, and this difference is independent of the stage of the disease; i.e., IgG4-dominant samples taken in the first acute episode, in a relapse and in remission have similar inhibitory capacities.

### Associations of Anti-ADAMTS13 IgG Levels and Subclass Distribution With Risk and Protective HLA-DR-DQ Haplotypes

The HLA-DR-DQ haplotypes were determined in 70 of the 81 patients. Forty-seven of the above patients carried risk (DR11-DQ3 or DR15-DQ6) and nine of them protective (DR7-DQ2 or DR13-DQ6) haplotypes, two of these patients carried both. The proportion of anti-ADAMTS13 IgG subclasses did not differ between patients carrying and not carrying either risk or protective haplotypes.

**Figure 7:**
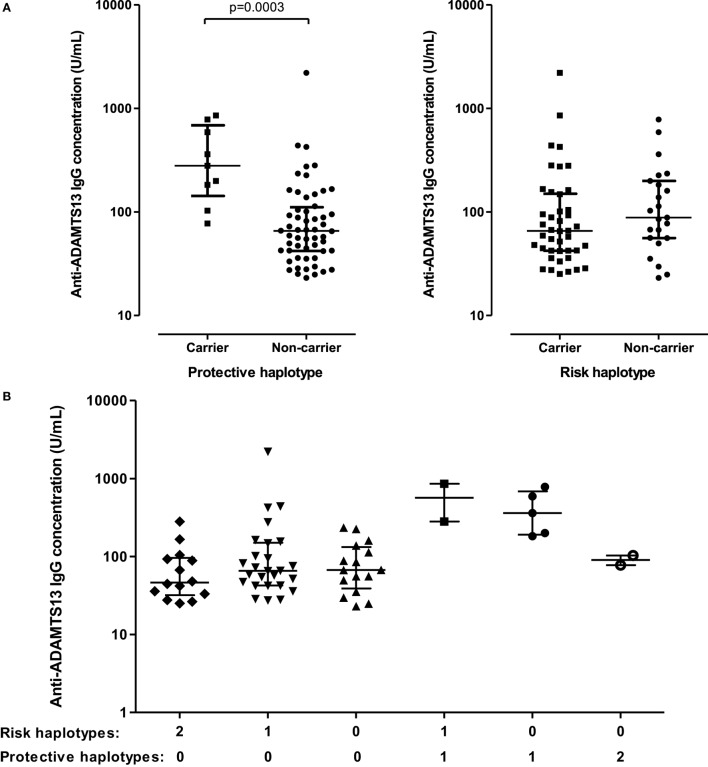

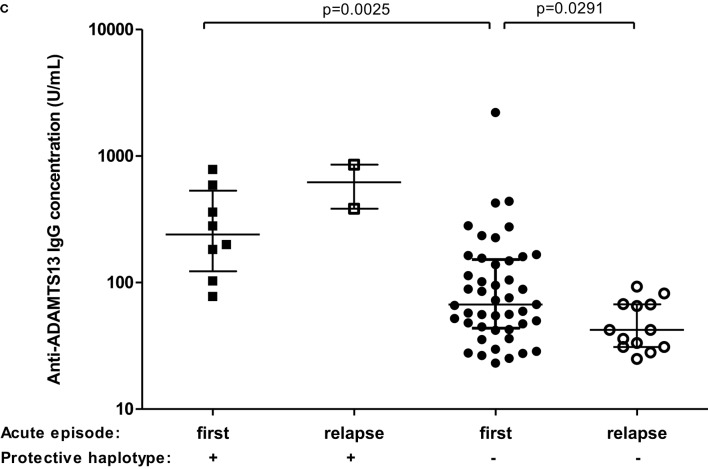
Anti-ADAMTS13 IgG levels in patients carrying or not carrying protective (DR7-DQ2 or DR13-DQ6) or risk (DR11-DQ3 or DR15-DQ6) HLA-DR-DQ haplotypes. Medians and interquartile ranges are shown. **(A,B)** Anti-ADAMTS13 IgG levels in the first available acute sample (either from the first acute episode or from relapse) of each patient with available HLA-DR-DQ data. The numbers below panel **(B)** show the number of risk or protective haplotypes carried by the patient. **(C)** Samples from the first acute episode and from relapse. +: protective haplotype carrier, −: not carrying protective haplotypes.

However, when comparing the first available acute sample of each patient with known HLA-DR-DQ haplotypes, we found that the concentration of anti-ADAMTS13 autoantibodies were higher in patients carrying, than in those not carrying protective haplotypes [280.4 (142.8–687.0) vs. 65.7 (42.1–111.4) U/mL, *p* = 0.0003] (Figure 7A). Conversely, no difference could be observed between patients carrying and not carrying risk haplotypes. There was no clear association between anti-ADAMTS13 IgG levels and the number of risk or protective haplotypes carried (gene dosage) (Figure 7B).

Comparing samples from the first acute episode, we found that the anti-ADAMTS13 IgG levels were higher in patients carrying protective haplotypes than in those not carrying protective haplotypes [240.2 (122.9–533.1) vs. 67.1 (43.6–152.0) U/mL, *p* = 0.0025] (Figure 7C). Interestingly, patients not carrying protective haplotypes had lower total anti-ADAMTS13 IgG levels during relapse than during the first acute episode [42.3 (31.0–67.4) vs. 67.1 (43.6–152.0) U/mL, *p* = 0.0291]. No such trend could be observed in patients carrying protective haplotypes.

## Discussion

In order to investigate the changes in the antibody response against ADAMTS13 during the course of TTP, we analyzed the amount, subclass distribution, and inhibitory potential of anti-ADAMTS13 autoantibodies in 101 samples of 81 TTP patients, drawn in different stages of the disease.

We found that most anti-ADAMTS13 IgG autoantibodies belonged to the IgG1 and IgG4 subclasses, which were present in most samples. Whereas almost half of the samples drawn during the first acute episode were IgG1-dominant, all samples taken during or following a relapse were IgG4 dominant. The inhibitory potential of samples increased parallel to the change to IgG4-dominance, and correlated with the levels of anti-ADAMTS13 IgG4. Patients carrying protective DR7-DQ2 and DR13-DQ6 haplotypes had higher total anti-ADAMTS13 IgG levels compared to those not carrying protective haplotypes. Patients who did not carry protective haplotypes had lower total anti-ADAMTS13 IgG levels during relapse than during the first acute episode.

During the inclusion period, 113 patients were diagnosed with acquired idiopathic TTP in our laboratory, 81 of the above patients had altogether 101 appropriate samples for the determination of anti-ADAMTS13 IgG levels and subclass distribution. The presence and amount of anti-ADAMTS13 inhibitors were tested in 97 of the above samples, and the specific inhibitory potential of anti-ADAMTS13 antibodies were determined in 49 of them. HLA-DR-DQ haplotypes were determined in 70 TTP patients. The age and gender distribution and the mortality and relapse rate of patients in our cohort are consistent with data from the literature (20, 29).

In agreement with previous studies (16–19), we found that the anti-ADAMTS13 IgG autoantibodies belonged primarily to the IgG1 and IgG4 subclasses, which were present in most samples, whereas IgG2 and IgG3 were present only in smaller subsets of samples, and only in lower concentrations. In contrast with previous observations (16), we found that the proportions, but not the absolute amounts of IgG1 and IgG4 showed an inverse correlation. On the contrary, concentrations of IgG1 and IgG4 were positively correlated in our study, possibly because antibody levels were more variable than the proportions of the subclasses, so samples with high or low total anti-ADAMTS13 concentrations contained high or low concentrations of both subclasses, respectively. On the other hand, the levels of IgG3, found mainly in IgG1-dominant samples, showed a strong inverse correlation with IgG4 levels, supporting a difference in the polarity of the immune response.

The IgG1 and IgG3 subclasses are potent inducers of inflammation as they can effectively bind to Fcγ receptors and can activate the classical pathway of the complement system. IgG4, on the contrary, tends to be anti-inflammatory, as it cannot activate the complement system *via* the classical pathway, and binds Fcγ molecules with low affinity (30, 31). Moreover, IgG4 molecules are capable of exchanging one heavy chain and the associated light chain with other IgG4 molecules, resulting in bispecific, functionally monovalent antibodies (32). Because of their functional monovalency, they are unable to form large immune complexes, which further attenuate their inflammatory property (31, 33). In spite of their low inflammatory potential, IgG4 antibodies are involved in the pathogenesis of numerous autoimmune disorders (34, 35). IgG4 antibodies are often produced following prolonged antigenic stimulation (36). Accordingly, IgG4 seems to emerge as the dominant subclass in the ADAMTS13-specific antibody response in later stages of acquired TTP.

Our study provides independent validation of the polarized nature of autoantibody development to ADAMTS13 in TTP. In agreement with the results of Ferrari et al. (16), whereas IgG1 was the predominant subclass in almost half of the samples drawn during the first acute episode, all deficient samples drawn during or following a relapse were IgG4 dominant (Table 1; Figure 3). Moreover, all four patients who had unexplained events of thrombocytopenia before the diagnosis of TTP, corresponding to subclinical episodes of the disease, had IgG4-dominant anti-ADAMTS13 antibody response at the time of the first clinical acute episode. Besides the above comparison of independent samples, results of patients with multiple samples further support this observation: IgG4 was the dominant subclass in all samples taken in relapse, or in remission following a relapse. Two of the four patients with initially IgG1-dominant samples had IgG4-dominant samples already in remission following the first episode (neither of them has relapsed to date). Contrarily, none of the patients with initially IgG4-dominant samples had subsequent IgG1-dominant samples. One of them had an IgG3-dominant sample, however, with normal ADAMTS13 activity, which suggests the disappearance or ceased antibody production of the IgG4-producing clones (Figure 4).

The observation on the restricted clonality, i.e., the lack of IgG1-dominant immune response to ADAMTS13 in relapse of TTP, is intriguing. The antibody response against ADAMTS13 in TTP is a polyclonal one, as was shown by cloning ADAMTS13-specific B cells (37–40). A single B cell clone produces antibodies of exclusively one subclass, thus, our observation that most patients have anti-ADAMTS13 IgG of multiple subclasses provides further evidence for the polyclonality of the antibody response.

In this study, we provide evidence of an association between the subclass distribution and inhibitory potential of anti-ADAMTS13 antibodies. IgG4 levels showed a strong correlation with the inhibitory potential of the sample (Figure 5), the correlation was already present at the time of the first acute episode. In addition, direct, head-to-head comparison of samples with or without IgG4-dominant anti-ADAMTS13 antibody response showed remarkable differences: equal amounts of IgG4-dominant ADAMTS13 antibodies had increased inhibitory potential, when compared to non-IgG4-dominant autoantibodies (Figure 7). This difference was unrelated to disease course, since the specific inhibitory potentials of IgG4-dominant samples from different disease stages showed no difference. Whether the increase of the inhibitory potential of IgG4 autoantibodies is a consequence of the altered subclass proportions (associated with different Fcγ receptor binding, half-life, or heavy-chain exchange), or a consequence of other parallel mechanisms, like affinity maturation (resulting in increased affinity, avidity, and specificity), cannot be unequivocally determined from these data.

We found that total anti-ADAMTS13 IgG levels were lower during a relapse than during the first acute episode, this difference was statistically significant in the subset of patients who did not carry protective haplotypes (Figure 7C). Regarding the increase of the inhibitory potential of anti-ADAMTS13 antibodies parallel to the switch to IgG4-dominance prior to a relapse, this finding suggests that, because of the stronger inhibitory capacity of autoantibodies, lower IgG concentrations may be sufficient to cause severe ADAMTS13 deficiency resulting in increased risk of a TTP episode. The lower anti-ADAMTS13 IgG levels during a relapse, together with the lower proportion of complement-activating and Fcγ receptor-binding IgG subclasses, may contribute to the less severe clinical picture seen in relapse compared to the first acute episode (41).

Interestingly, total anti-ADAMTS13 IgG levels were higher in patients carrying protective HLA-DR-DQ haplotypes (DR7-DQ2, DR13-DQ6). There was no difference between patients carrying and not carrying risk haplotypes (DR11-DQ3, DR15-DQ6), though. Whereas there are accumulating data about the role of the DRB1*11 risk allele in the pathogenesis of TTP through efficient presentation of certain ADAMTS13-derived peptides by the DR11 molecule (22, 23, 42, 43), the mechanism by which the protective haplotypes lower the risk of developing TTP is to date obscure. There was no difference in the proportion of the IgG subclasses between groups of patients carrying or not carrying risk or protective haplotypes. Whether the samples of patients carrying protective haplotypes have weaker inhibitory capacities is an intriguing question; however, the low number of patients carrying protective haplotypes precluded statistical analysis in this study.

There are a number of strengths and potential limitations of this study. We determined the amount, subclass-distribution, and inhibitory potential of anti-ADAMTS13 IgG antibodies in anti-ADAMTS13 IgG-positive samples of a large and homogenous group of ADAMTS13-deficient acquired TTP patients. We compared the above parameters between disease stages and between patients carrying or not carrying risk or protective HLA-DR-DQ haplotypes. We found that whereas IgG1 was the dominant subclass in almost half of the patients during the first acute episode, IgG4 became the dominant subclass in all patients by the time of the first relapse. Interestingly, not a single relapse episode was linked to IgG1-dominant anti-ADAMTS13 autoantibodies. The inhibitory potential of the samples correlated with the anti-ADAMTS13 IgG4 levels, and we provided direct evidence that IgG4 autoantibodies have stronger inhibitory potentials than non-IgG4 ones. No differences were observed in the subclass distribution of patients with different HLA-DR-DQ backgrounds, but the total anti-ADAMTS13 IgG levels were markedly higher in patients carrying protective haplotypes than in those not carrying them. Potential limitations that may apply to this study include the descriptive, retrospective nature of the design, and the lack of prior sample size calculation. In addition, important subgroups, for example relapsing patients with available samples from acute disease, are limited in number. Taken all of these aspects together, our results provide further observational and experimental evidence for the presence of evolving immune response to ADAMTS13 in TTP.

These observations may, therefore, have important consequences on the clinical management of acquired TTP. First, early and strong immunosuppressive therapy, with the goal to eradicate the ADAMTS13 inhibitor-producing B cell clones, seems to be indicated to avoid later affinity maturation and isotype switching. In addition, the analysis of anti-ADAMTS13 subclasses may provide helpful information on risk of development of ADAMTS13 deficiency, hence on follow-up protocol. Finally, it seems to be rational to study immunomodulating or cytokine-targeting therapies in the future to prevent the development of matured, high affinity inhibitors to ADAMTS13 in TTP.

## Ethics Statement

The present study was conducted in accordance with the recommendations of the Declaration of Helsinki and the Human Research Ethics Committee. The protocol was approved by the Human Research Ethics Committee. All subjects gave written informed consent in accordance with the Declaration of Helsinki.

## Author Contributions

Research was designed by ZP and GS. Patient samples and data were provided by MR, KR, and PF. Anti-ADAMTS13 IgG levels and subclass distribution were determined by GS. HLA analysis was performed by DI, AS, AT, and ÁS. The first draft was written by GS, with contributions by ZP. Data were analyzed, interpreted, and the manuscript was critically reviewed and approved by all authors. Study was supervised by ZP.

## Conflict of Interest Statement

The authors declare that the research was conducted in the absence of any commercial or financial relationships that could be construed as a potential conflict of interest.

## References

[1] Kremer Hovinga JA Heeb SR Skowronska M Schaller M Pathophysiology of thrombotic thrombocytopenic purpura and hemolytic uremic syndrome. J Thromb Haemost (2018) 16(4):618–29. 10.1111/jth.13956 29356300

[2] Moake JL Rudy CK Troll JH Weinstein MJ Colannino NM Azocar J Unusually large plasma factor VIII:von Willebrand factor multimers in chronic relapsing thrombotic thrombocytopenic purpura. N Engl J Med (1982) 307(23):1432–5. 10.1056/NEJM198212023072306 6813740

[3] Hosler GA Cusumano AM Hutchins GM. Thrombotic thrombocytopenic purpura and hemolytic uremic syndrome are distinct pathologic entities. A review of 56 autopsy cases. Arch Pathol Lab Med (2003) 127(7):834–9. 10.1043/1543-2165(2003)127<834:TTPAHU>2.0.CO;2 12823037

[4] Fujikawa K Suzuki H McMullen B Chung D. Purification of human von Willebrand factor-cleaving protease and its identification as a new member of the metalloproteinase family. Blood (2001) 98(6):1662–6. 10.1182/blood.V98.6.1662 11535495

[5] Zheng X Chung D Takayama TK Majerus EM Sadler JE Fujikawa K Structure of von Willebrand factor-cleaving protease (ADAMTS13), a metalloprotease involved in thrombotic thrombocytopenic purpura. J Biol Chem (2001) 276(44):41059–63. 10.1074/jbc.C100515200 11557746

[6] Furlan M Robles R Solenthaler M Wassmer M Sandoz P Lammle B. Deficient activity of von Willebrand factor-cleaving protease in chronic relapsing thrombotic thrombocytopenic purpura. Blood (1997) 89(9):3097–103. 9129011

[7] Levy GG Nichols WC Lian EC Foroud T McClintick JN McGee BM Mutations in a member of the ADAMTS gene family cause thrombotic thrombocytopenic purpura. Nature (2001) 413(6855):488–94. 10.1038/35097008 11586351

[8] Furlan M Robles R Galbusera M Remuzzi G Kyrle PA Brenner B von Willebrand factor-cleaving protease in thrombotic thrombocytopenic purpura and the hemolytic-uremic syndrome. N Engl J Med (1998) 339(22):1578–84. 10.1056/NEJM199811263392202 9828245

[9] Tsai HM Lian EC. Antibodies to von Willebrand factor-cleaving protease in acute thrombotic thrombocytopenic purpura. N Engl J Med (1998) 339(22):1585–94. 10.1056/NEJM199811263392203 9828246 PMC3159001

[10] Scheiflinger F Knobl P Trattner B Plaimauer B Mohr G Dockal M Nonneutralizing IgM and IgG antibodies to von Willebrand factor-cleaving protease (ADAMTS-13) in a patient with thrombotic thrombocytopenic purpura. Blood (2003) 102(9):3241–3. 10.1182/blood-2003-05-1616 12855569

[11] Feys HB Liu F Dong N Pareyn I Vauterin S Vandeputte N ADAMTS-13 plasma level determination uncovers antigen absence in acquired thrombotic thrombocytopenic purpura and ethnic differences. J Thromb Haemost (2006) 4(5):955–62. 10.1111/j.1538-7836.2006.01833.x 16689741

[12] Shelat SG Smith P Ai J Zheng XL. Inhibitory autoantibodies against ADAMTS-13 in patients with thrombotic thrombocytopenic purpura bind ADAMTS-13 protease and may accelerate its clearance in vivo. J Thromb Haemost (2006) 4(8):1707–17. 10.1111/j.1538-7836.2006.02025.x 16879212 PMC2577225

[13] Thomas MR de Groot R Scully MA Crawley JT. Pathogenicity of Anti-ADAMTS13 autoantibodies in acquired thrombotic thrombocytopenic purpura. EBioMedicine (2015) 2(8):942–52. 10.1016/j.ebiom.2015.06.007 26425702 PMC4563118

[14] Rieger M Mannucci PM Kremer Hovinga JA Herzog A Gerstenbauer G Konetschny C ADAMTS13 autoantibodies in patients with thrombotic microangiopathies and other immunomediated diseases. Blood (2005) 106(4):1262–7. 10.1182/blood-2004-11-4490 15890682

[15] Ferrari S Scheiflinger F Rieger M Mudde G Wolf M Coppo P Prognostic value of anti-ADAMTS 13 antibody features (Ig isotype, titer, and inhibitory effect) in a cohort of 35 adult French patients undergoing a first episode of thrombotic microangiopathy with undetectable ADAMTS 13 activity. Blood (2007) 109(7):2815–22. 10.1182/blood-2006-02-006064 17164349

[16] Ferrari S Mudde GC Rieger M Veyradier A Kremer Hovinga JA Scheiflinger F. IgG subclass distribution of anti-ADAMTS13 antibodies in patients with acquired thrombotic thrombocytopenic purpura. J Thromb Haemost (2009) 7(10):1703–10. 10.1111/j.1538-7836.2009.03568.x 19682238

[17] Bettoni G Palla R Valsecchi C Consonni D Lotta LA Trisolini SM ADAMTS-13 activity and autoantibodies classes and subclasses as prognostic predictors in acquired thrombotic thrombocytopenic purpura. J Thromb Haemost (2012) 10(8):1556–65. 10.1111/j.1538-7836.2012.04808.x 22672482

[18] Ferrari S Palavra K Gruber B Kremer Hovinga JA Knobl P Caron C Persistence of circulating ADAMTS13-specific immune complexes in patients with acquired thrombotic thrombocytopenic purpura. Haematologica (2014) 99(4):779–87. 10.3324/haematol.2013.094151 24241492 PMC3971089

[19] Pos W Sorvillo N Fijnheer R Feys HB Kaijen PH Vidarsson G Residues Arg568 and Phe592 contribute to an antigenic surface for anti-ADAMTS13 antibodies in the spacer domain. Haematologica (2011) 96(11):1670–7. 10.3324/haematol.2010.036327 21712537 PMC3208685

[20] Kremer Hovinga JA Vesely SK Terrell DR Lammle B George JN. Survival and relapse in patients with thrombotic thrombocytopenic purpura. Blood (2010) 115(8):1500–11; quiz 662. 10.1182/blood-2009-09-243790 20032506

[21] Peyvandi F Lavoretano S Palla R Feys HB Vanhoorelbeke K Battaglioli T ADAMTS13 and anti-ADAMTS13 antibodies as markers for recurrence of acquired thrombotic thrombocytopenic purpura during remission. Haematologica (2008) 93(2):232–9. 10.3324/haematol.11739 18223285

[22] Scully M Brown J Patel R McDonald V Brown CJ Machin S. Human leukocyte antigen association in idiopathic thrombotic thrombocytopenic purpura: evidence for an immunogenetic link. J Thromb Haemost (2010) 8(2):257–62. 10.1111/j.1538-7836.2009.03692.x 19922436

[23] Coppo P Busson M Veyradier A Wynckel A Poullin P Azoulay E HLA-DRB1*11: a strong risk factor for acquired severe ADAMTS13 deficiency-related idiopathic thrombotic thrombocytopenic purpura in Caucasians. J Thromb Haemost (2010) 8(4):856–9. 10.1111/j.1538-7836.2010.03772.x 20141578

[24] John ML Hitzler W Scharrer I. The role of human leukocyte antigens as predisposing and/or protective factors in patients with idiopathic thrombotic thrombocytopenic purpura. Ann Hematol (2012) 91(4):507–10. 10.1007/s00277-011-1384-z 22203269

[25] Sinkovits G Szilagyi A Farkas P Inotai D Szilvasi A Tordai A The role of human leukocyte antigen DRB1-DQB1 haplotypes in the susceptibility to acquired idiopathic thrombotic thrombocytopenic purpura. Hum Immunol (2017) 78(2):80–7. 10.1016/j.humimm.2016.11.005 27866840

[26] Gombos T Mako V Cervenak L Papassotiriou J Kunde J Harsfalvi J Levels of von Willebrand factor antigen and von Willebrand factor cleaving protease (ADAMTS13) activity predict clinical events in chronic heart failure. Thromb Haemost (2009) 102(3):573–80. 10.1160/TH09-01-0036 19718479

[27] Stephens M Smith NJ Donnelly P. A new statistical method for haplotype reconstruction from population data. Am J Hum Genet (2001) 68(4):978–89. 10.1086/319501 11254454 PMC1275651

[28] Stephens M Scheet P. Accounting for decay of linkage disequilibrium in haplotype inference and missing-data imputation. Am J Hum Genet (2005) 76(3):449–62. 10.1086/428594 15700229 PMC1196397

[29] Coppo P Schwarzinger M Buffet M Wynckel A Clabault K Presne C Predictive features of severe acquired ADAMTS13 deficiency in idiopathic thrombotic microangiopathies: the French TMA reference center experience. PLoS One (2010) 5(4):e10208. 10.1371/journal.pone.0010208 20436664 PMC2859048

[30] Bruggemann M Williams GT Bindon CI Clark MR Walker MR Jefferis R Comparison of the effector functions of human immunoglobulins using a matched set of chimeric antibodies. J Exp Med (1987) 166(5):1351–61. 10.1084/jem.166.5.1351 3500259 PMC2189658

[31] Bruhns P Iannascoli B England P Mancardi DA Fernandez N Jorieux S Specificity and affinity of human Fcgamma receptors and their polymorphic variants for human IgG subclasses. Blood (2009) 113(16):3716–25. 10.1182/blood-2008-09-179754 19018092

[32] van der Zee JS van Swieten P Aalberse RC. Serologic aspects of IgG4 antibodies. II. IgG4 antibodies form small, nonprecipitating immune complexes due to functional monovalency. J Immunol (1986) 137(11):3566–71. 3782791

[33] van der Neut Kolfschoten M Schuurman J Losen M Bleeker WK Martinez-Martinez P Vermeulen E Anti-inflammatory activity of human IgG4 antibodies by dynamic Fab arm exchange. Science (2007) 317(5844):1554–7. 10.1126/science.1144603 17872445

[34] Ludwig RJ Vanhoorelbeke K Leypoldt F Kaya Z Bieber K McLachlan SM Mechanisms of autoantibody-induced pathology. Front Immunol (2017) 8:603. 10.3389/fimmu.2017.00603 28620373 PMC5449453

[35] Koneczny I. A new classification system for IgG4 autoantibodies. Front Immunol (2018) 9:97. 10.3389/fimmu.2018.00097 29483905 PMC5816565

[36] Aalberse RC van der Gaag R van Leeuwen J. Serologic aspects of IgG4 antibodies. I. Prolonged immunization results in an IgG4-restricted response. J Immunol (1983) 130(2):722–6. 6600252

[37] Luken BM Kaijen PH Turenhout EA Kremer Hovinga JA van Mourik JA Fijnheer R Multiple B-cell clones producing antibodies directed to the spacer and disintegrin/thrombospondin type-1 repeat 1 (TSP1) of ADAMTS13 in a patient with acquired thrombotic thrombocytopenic purpura. J Thromb Haemost (2006) 4(11):2355–64. 10.1111/j.1538-7836.2006.02164.x 16898953

[38] Pos W Luken BM Kremer Hovinga JA Turenhout EA Scheiflinger F Dong JF VH1-69 germline encoded antibodies directed towards ADAMTS13 in patients with acquired thrombotic thrombocytopenic purpura. J Thromb Haemost (2009) 7(3):421–8. 10.1111/j.1538-7836.2008.03250.x 19054323

[39] Schaller M Vogel M Kentouche K Lammle B Kremer Hovinga JA. The splenic autoimmune response to ADAMTS13 in thrombotic thrombocytopenic purpura contains recurrent antigen-binding CDR3 motifs. Blood (2014) 124(23):3469–79. 10.1182/blood-2014-04-561142 25261198

[40] Ostertag EM Kacir S Thiboutot M Gulendran G Zheng XL Cines DB ADAMTS13 autoantibodies cloned from patients with acquired thrombotic thrombocytopenic purpura: 1. Structural and functional characterization in vitro. Transfusion (2016) 56(7):1763–74. 10.1111/trf.13584 27040144 PMC4938786

[41] Page EE Kremer Hovinga JA Terrell DR Vesely SK George JN. Thrombotic thrombocytopenic purpura: diagnostic criteria, clinical features, and long-term outcomes from 1995 through 2015. Blood Adv (2017) 1(10):590–600. 10.1182/bloodadvances.2017005124 29296701 PMC5728353

[42] Sorvillo N van Haren SD Kaijen PH ten Brinke A Fijnheer R Meijer AB Preferential HLA-DRB1*11-dependent presentation of CUB2-derived peptides by ADAMTS13-pulsed dendritic cells. Blood (2013) 121(17):3502–10. 10.1182/blood-2012-09-456780 23372163

[43] Verbij FC Turksma AW de Heij F Kaijen P Lardy N Fijnheer R CD4+ T cells from patients with acquired thrombotic thrombocytopenic purpura recognize CUB2 domain-derived peptides. Blood (2016) 127(12):1606–9. 10.1182/blood-2015-10-668053 26747250

