# Supplementary material for: Concentration and Subclass Distribution of Anti-ADAMTS13 IgG Autoantibodies in Different Stages of Acquired Idiopathic Thrombotic Thrombocytopenic Purpura
Source: Front Immunol. 2018 Jul 16;9:1646. doi: 10.3389/fimmu.2018.01646 (PMC6054987; doi:10.3389/fimmu.2018.01646)
Supplement: Supplementary file 1 [file Data_Sheet_1.PDF]

Supplementary Material

Concentration and Subclass Distribution of Anti-ADAMTS13 IgG Autoantibodies in Different Stages of Acquired Idiopathic Thrombotic Thrombocytopenic Purpura

György Sinkovits*, Ágnes Szilágyi, Péter Farkas, Dóra Inotai, Anikó Szilvási, Attila Tordai, Katalin Rázsó, Marienn Réti, Zoltán Prohászka

***Correspondence:** György Sinkovits, gysinkovits@gmail.com

1 Development and design of an ELISA method to determine the proportions and concentrations of IgG1, IgG2, IgG3 and IgG4 anti-ADAMTS13 autoantibodies

1.1 Introduction, outline

Our aim was to develop an ELISA-based method which is suitable for the exact determination of the proportions of IgG subclasses of anti-ADAMTS13 antibodies.

The basic principle of the immunoassay is to incubate diluted patient samples in wells coated with recombinant ADAMTS13, and to selectively detect each IgG subclass of the bound anti-ADAMTS13 antibodies by using subclass-specific secondary antibodies. The enzyme (horseradish peroxidase) coupled to the secondary antibodies converts the chromogenic substrate (tetramethylbenzidine, TMB) producing the measured signal (optical density, OD).

To solve the problem of the different affinities of the various subclass-specific secondary antibodies and of the potentially different activities of the conjugated enzymes, subclass-specific calibrators were applied in each measurement. Optical density values obtained in this system were converted to bound antibody amounts. These are directly comparable between distinct subclasses; therefore, the subclass distribution of a given patient's sample can be exactly determined.

1.2 Materials

The following materials were used:

<u>Microtiter plates:</u>

- Polystyrene 96 well flat-bottom clear microplate,
  Greiner Bio-One International GmbH, Kremsmünster, Austria; Ref. No.: 655101
- Microplate (8-well test strips) coated with recombinant ADAMTS13,
  Technozym® ADAMTS-13 INH kit, Technoclone GmbH, Vienna, Austria; Ref. No.: 5450401

<u>Purified, full length human IgG1, IgG2, IgG3, and IgG4 proteins</u>

Abcam plc, Cambridge, UK,

Catalogue numbers:

- IgG1: ab90283
- IgG2: ab90284
- IgG3: ab118426
- IgG4: ab90286

Subclass-specific secondary antibodies:

Mouse anti-human antibodies against the Fc region,
Southern Biotech, Birmingham, AL, USA
Catalogue numbers:

- anti-IgG1: 9054-05
- anti-IgG2: 9060-05
- anti-IgG3: 9210-05
- anti-IgG4: 9200-05

Buffers:

- Coating buffer: Sodium-bicarbonate buffer (pH 9.8)
- Blocking buffer: Phosphate buffered saline (PBS, pH 7.4) containing 0.5% gelatine
- Washing and dilution buffers:
  Technozym® ADAMTS-13 INH kit, Technoclone GmbH, Vienna, Austria; Ref. No.: 5450401

Chromogenic substrate:

- Tetramethylbenzidine (TMB):
  Novex® TMB by life technologies, Frederick, MD, USA; Ref. No.: 00-2023

1.3 Specificity and optimal concentrations of the subclass-specific secondary antibodies

To test the subclass-specificity of the secondary antibodies, we coated seven microtiter plates with 100 ng/well of human IgM, IgG, IgG1, IgG2, IgG3, IgG4, and bicarbonate buffer (uncoated), respectively. After blocking, we incubated each row with increasing dilutions of anti-IgM, anti-IgG, anti-IgG1, anti-IgG2, anti-IgG3, anti-IgG4, anti-κ-chain antibodies, and dilution buffer, respectively. We developed each plate for 8 minutes with OPD. **Supplementary Figure 1** shows the binding of the subclass-specific secondary antibodies to the antibodies of distinct subclasses.

As it can be seen on **Supplementary Figure 1**, all subclass-specific antibodies bind to their respective subclass, and they do not bind other subclasses significantly.

Next, with further measurements using TMB as a substrate, we selected the optimal concentrations of the secondary antibodies, i.e. the lowest concentrations with detectable signal strengths and antibody excess.

The dilutions used for the assays:

- IgG1: 1:1000
- IgG2: 1:1000

- IgG3: 1:10000
- IgG4: 1:5000

1.4 Determination of ideal developing times for each subclass during the chromogenic substrate reaction

In spite of the higher dilutions of anti-IgG3 and anti-IgG4, these secondary antibodies produced stronger signals, i.e. higher OD values, than anti-IgG1 and IgG2 antibodies, when developed for the same amount of time. Therefore, to receive optimal results, different development times had to be used for each subclass during the substrate reaction phase. We found that the optimal development times were the following:

- IgG1 – anti-IgG1: 8 minutes
- IgG2 – anti-IgG2: 5 minutes
- IgG3 – anti-IgG3: 1.5 minutes
- IgG4 – anti-IgG4: 1.5 minutes

The difference in developing times did not bias the measurement, because the optical density produced by a sample tested for a certain subclass was compared to the calibrators of the same subclass, which were developed for the same length of time (described in '1.8. Calculation of the amounts of bound antibodies'). The test wells and the calibrator wells corresponding to the same subclass were always developed for exactly the same time length.

1.5 Preparation of calibrator plates

We added 100 μL of bicarbonate buffer (pH 9.8) to each well of a polystyrene 96 well flat-bottom clear microplate (Greiner), and prepared 2 μg/mL dilutions of each human antibody subclass. Then we added 100 uL of these antibody dilutions to the first wells of two columns for each subclass:

- IgG1: columns 1 and 2
- IgG2: columns 3 and 4
- IgG3: columns 5 and 6
- IgG4: columns 7 and 8

We mixed the wells and created 1:2 dilution series in the respective columns, leaving the last row blank. Thus, we received two parallel dilution series from 1 μg/mL (100 ng/well) to 15.625 ng/mL (1.56 ng/well) and 0 μg/mL (0 ng/well) in case of each subclass. (The schematic layout is shown on Supplementary Figure 3.)

The wells were incubated overnight at 4°C.

To confirm the efficacy of coating in case of all human IgG subclasses, we performed preliminary measurements, in which, after overnight incubation at 4°C, we pipetted the content of the wells to the respective wells of another microtiter plate, and repeated the overnight incubation in the second plate. After blocking, both plates were incubated by the respective subclass-specific secondary anti-human IgG antibodies for 1 hour at room temperature. The plates were then washed six times and developed with TMB. **Supplementary Figure 2** shows the amounts of antibodies detected in the wells of the second plate, interpolated from standard curves generated from the data of the original plate. The signal strengths on the second plate were below 1% of those of the original plate in the case of each antibody subclass. These results indicate that less than 1% of the antibodies remained in

the soluble phase after the overnight incubation, thus more than 99% of them were bound to the wells of the first microtiter plate. Importantly, there was no significant difference between the binding efficiency of the human antibodies of different subclasses.

1.6 Selection of initial sample dilution factors

Initial dilution factors for the subclass determination depended on the total anti-ADAMTS13 IgG levels measured by the Technozym® ADAMTS-13 INH assay, based on the experiences of preliminary measurements. Samples with anti-ADAMTS13 IgG levels below 100 U/mL were diluted in 1:12.5, samples with antibody levels between 100-200 U/mL were diluted in 1:20, and samples with antibody levels above 200 U/mL were diluted in 1:50 or 1:100. Thus, the final working anti-ADAMTS13 antibody concentrations of the diluted patient samples varied between 2 U/mL and 10 U/mL.

As the optimal dilutions (anti-ADAMTS13 IgG concentrations) depended also on the proportions of antibody subclasses, these initial dilutions had to be modified in many cases. The criteria for repeating the measurements using higher or lower dilutions are detailed below (1.9. Evaluation of results).

Importantly, the concentrations used for the determination of each of the four subclasses were always the same.

1.7 Steps of the assay procedure to determine the proportions of IgG1, IgG2, IgG3, and IgG4 anti-ADAMTS13 autoantibodies

1. All reagents and plates were brought to room temperature.
2. The patient's samples were diluted in dilution buffer, as detailed above (1.6. Selection of initial sample dilution factors).
3. 100 μL/well of each diluted patient sample was added to four wells of the microtiter plate coated by recombinant ADAMTS13 (referred to as 'test plate'), one well corresponding to each subclass. (see **Supplementary Figure 3**)
4. Test plates were incubated for 1 hour at room temperature.
5. The calibrator plate (prepared as described in '1.5. Preparation of calibrator plates') was washed once with 200 μL/well washing buffer.
6. 110 μL/well blocking buffer was added to the calibrator plates.
7. The calibrator plate was incubated for 50 minutes at room temperature (while the samples were incubated on the test plate).
8. The calibrator plate was washed 3 times, and the test plate was washed 5 times with 200 μL/well washing buffer.
9. Subclass-specific conjugated secondary antibodies were added to respective columns of the test plate (each of them containing one well per sample), and to the wells of the calibration plate coated by the respective subclass. (see **Supplementary Figure 3**) (Dilutions of the secondary antibodies are described in '1.3. Specificity and optimal concentrations of the subclass-specific secondary antibodies')
10. Calibrator and test plates were incubated for 1 hour at room temperature.
11. Calibrator and test plates were washed 6 times with 200 μL/well washing buffer.
12. 100 μL of chromogenic substrate (TMB, undiluted) was added to each well.
13. Columns of test and calibrator plates corresponding to the same subclass were developed for the same amount of time. (1.4. Determination of ideal developing times for each subclass during the chromogenic substrate reaction)

14. Reaction was stopped by adding 50 µL/well 4M H_2SO_4.
15. Optical density was measured at a wavelength of 450 nm, with a reference wavelength of 620 nm.

1.8 Calculation of the amounts of bound antibodies

As it is shown on **Supplementary Figure 3**, each sample was given to four wells of the test plate, and each of these four wells was incubated with a different subclass-specific secondary antibody. Wells of the calibration plate coated with human antibodies of a given subclass were incubated with the subclass-specific secondary antibody against the respective subclass. All samples incubated with the same subclass-specific secondary antibody were developed for the same amount of time. Based on the optical densities and the known coating concentrations of the wells of the calibrator plate, we created calibration curves, using the statistical software GraphPad Prism 5 (GraphPad Softwares Inc., La Jolla, CA, USA) (**Supplementary Figure 4A**). The amounts of bound anti-ADAMTS13 antibodies of the respective IgG subclass were interpolated from these calibration curves. (**Supplementary Figure 4B**)

1.9 Evaluation of results

Next, we determined the upper limit of quantification (ULOQ) and the lower limit of detection (LLOD) based on the calibration curves. The ULOQ was defined as the highest point of the calibration curve with a slope above 0.02 OD/ng. This point was regarded as the cut-off between the near linear section of the curve, appropriate for interpolation, and the plateau section. The LLOD was defined as the concentration of the lowest calibrator with an OD value significantly different from that of the blank wells. If a value was below the LLOD of the corresponding subclass, it was regarded as zero. If a value was above the ULOQ of the respective subclass, its value could not be determined; therefore the measurement (i.e. the determination of all subclasses) had to be repeated using higher dilution ratios for all four subclasses.

To ensure the comparability of results throughout the set of measurements, we used patient samples with detectable amounts of subclasses IgG1, IgG3, and IgG4, as control samples. **Supplementary Table 1** shows the results of the control sample used in the third set of measurements. The low CV% values indicate good accuracy and reproducibility of our assay.

1.10 Calculation of the proportions of IgG subclasses

Thus, we determined the exact amounts (expressed in nanograms) of bound anti-ADAMTS13 antibodies of each IgG subclass. The proportion of anti-ADAMTS13 IgG subclasses in a patient sample were calculated according to the proportions of the bound antibodies. First, we calculated the total amount of bound antibodies by summing the amounts of bound antibodies of each subclass. The proportion of antibodies of a given subclass was then calculated by dividing the total amount of bound antibodies by the amount of bound antibodies of the respective subclass. (**Supplementary Figure 5A**)

1.11 Calculation of absolute concentrations of IgG subclasses

The amount of bound antibodies, used for the determination of subclass proportions, was not appropriate for the direct determination of the absolute concentrations of anti-ADAMTS13 antibodies of certain IgG subclasses, though.

Because of the different affinities of distinct subclass-specific secondary antibodies, the optimal dilutions for the determination of subclass proportions depended not only on the total anti-ADAMTS13 IgG concentrations, but also on the subclass distribution of the patient sample. (For example, a sample with anti-ADAMTS13 antibodies predominantly of the IgG1 subclass could be measured in higher concentrations than a sample with predominantly IgG4 antibodies, because of the relatively low affinity of anti-IgG1 secondary antibodies. Samples with predominantly IgG4 antibodies used in the same dilutions would have resulted in IgG4 values over the ULOQ because of the stronger signal produced by anti-IgG4 antibodies.) The comparison of samples measured in different dilutions could have lead to bias because of the matrix effect and the saturation kinetics of the antibody binding sites on the test wells.

Therefore, the absolute concentrations of the anti-ADAMTS13 IgG subclasses were calculated by multiplying the total anti-ADAMTS13 IgG concentrations (determined by the Technozym® ADAMTS-13 INH kit) by the proportions of IgG subclasses (calculated as described above). (**Supplementary Figure 5B**)

The relation between the absolute concentration of anti-ADAMTS13 antibodies of distinct IgG subclasses and the amount of bound antibodies multiplied by the dilution factors are shown on **Supplementary Figure 6**. The two measures correlate with each other, irrespective of IgG subclass.

1.12 Assay performance

We measured 14 negative samples (samples of 7 TTP patients with normal ADAMTS13 activity and of 7 healthy control subjects), and 14 samples of TTP patients with decreased but non-deficient (20-40%) ADAMTS13 activity. All 28 samples had anti-ADAMTS13 IgG levels below 15 U/mL by the Technozym ADAMTS-13 INH assay. All 14 negative samples produced negative results (below LLOD) for all subclasses. Two samples with decreased ADAMTS13 activities produced low positive results only in case of the IgG4 subclass, all other samples were negative. The above results suggest a high specificity of our assay.

The intra- and inter-assay coefficients of variation (CV%) of our assay are shown in **Supplementary Table 2**.

Supplementary Figure 1. Binding of the subclass-specific secondary antibodies to the antibodies of distinct subclasses. Mean and SD of two independent measurements are shown. Only the highest antibody concentrations (the range in which they were later used) are shown.

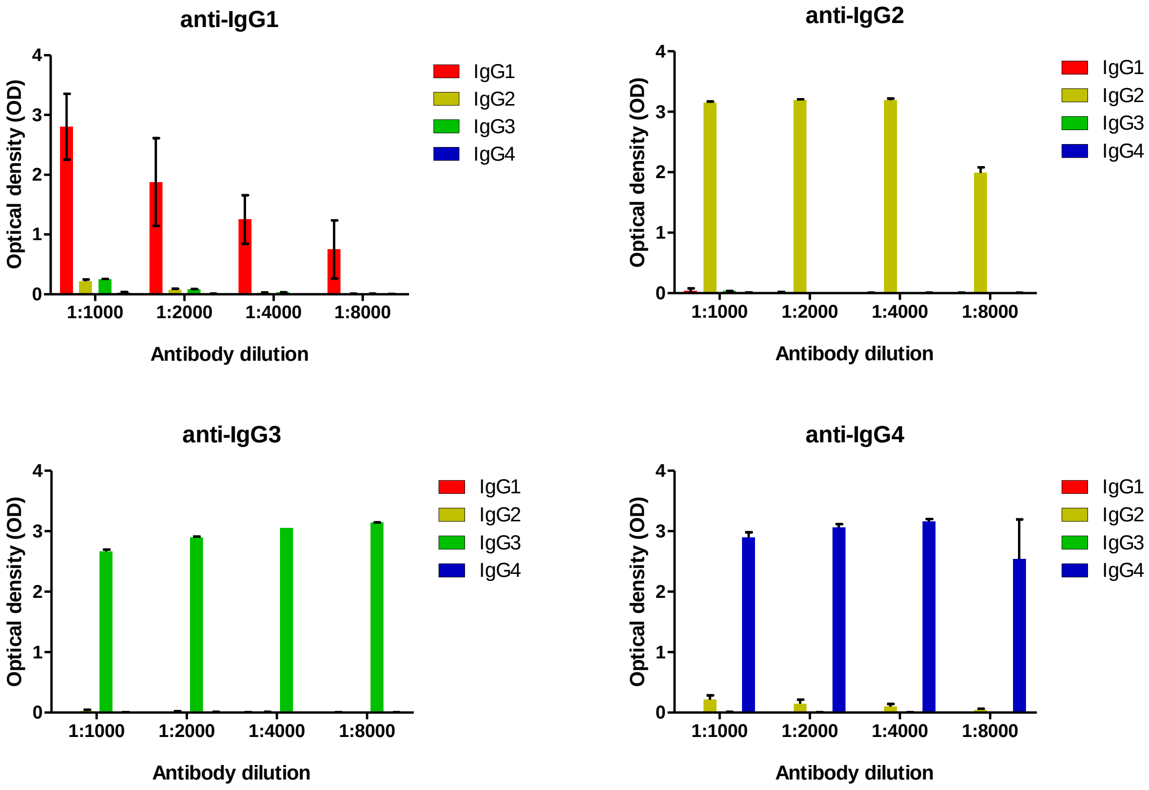

Supplementary Figure 2. Results of the preliminary measurement to confirm the coating efficiency. After coating, the contents of a microtiter plate (bicarbonate buffer with the unbound antibodies) were pipetted to respective wells of a new plate, where a second overnight incubation at 4°C was carried out. Both plates were blocked, incubated with anti-IgG secondary antibodies, and developed the same way. The figure shows the amount of antibodies detected in the second plate, indicating the amount of unbound antibodies during the coating of the first plate. (see the text for details)

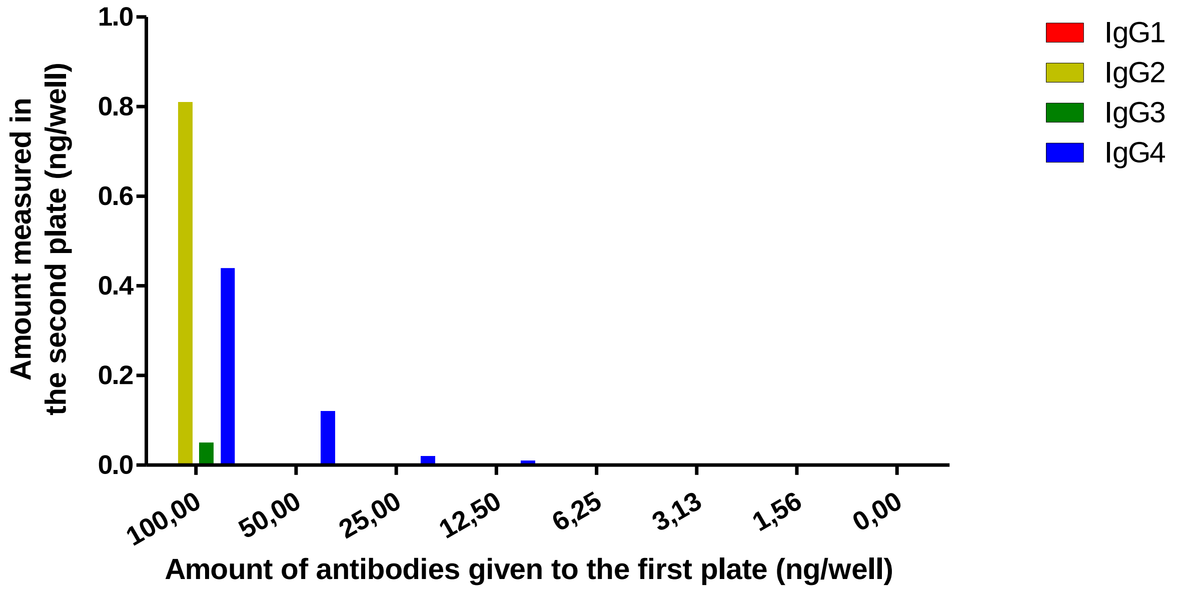

Supplementary Figure 3. Layouts of the calibrator and test plates during assay steps.

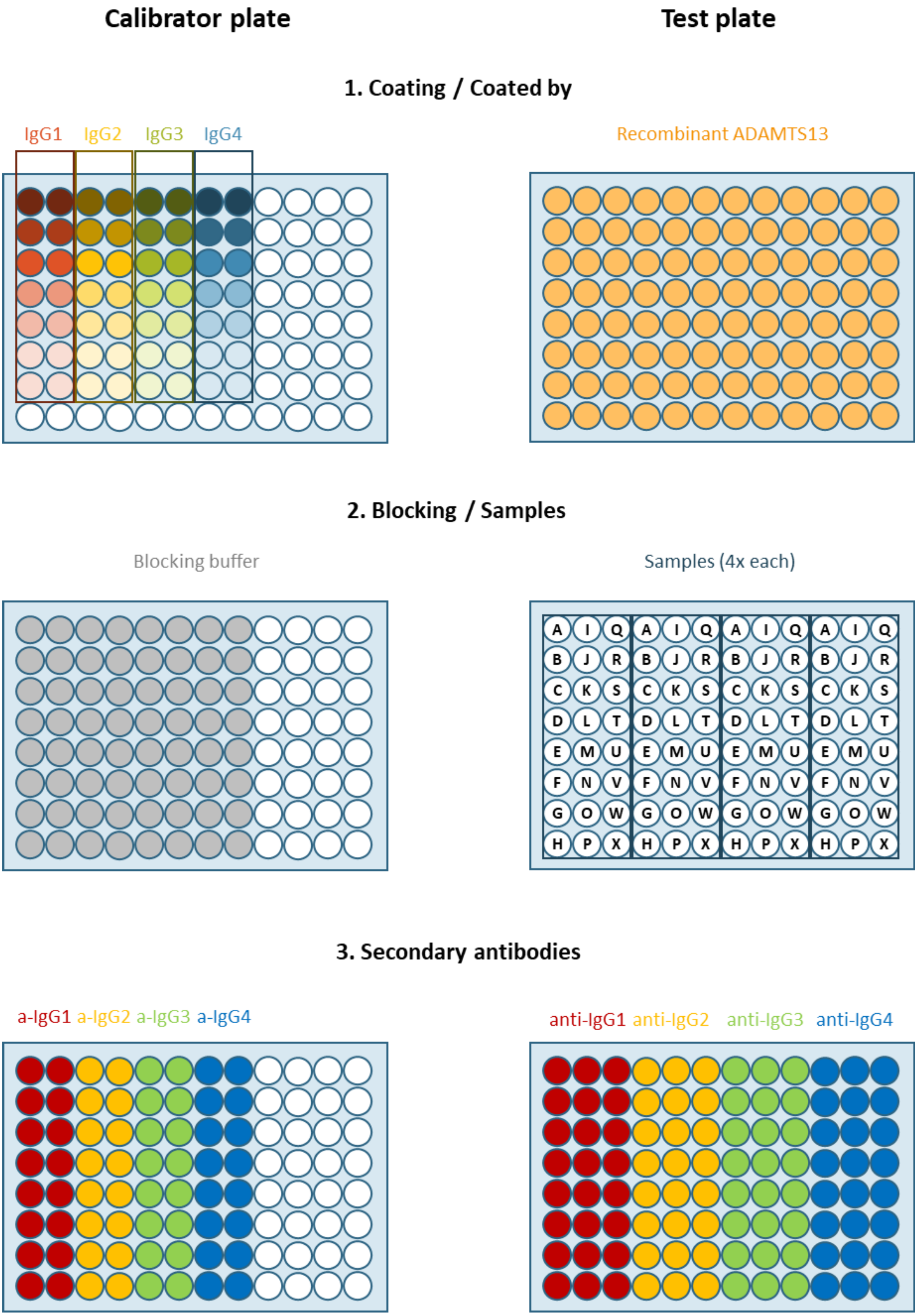

Supplementary Figure 4. Calibrator curves and interpolation. Supplementary figure 4A shows calibrator curves of a measurement generated from the OD values of wells coated with given amount of antibodies (on the calibration plate). Supplementary figure 4B shows an enlarged portion of the same curves, with solid lines representing the interpolation of the amount of anti-ADAMTS13 IgG subclasses of a patient sample. The calculated results of the sample are shown in the attached table. Dashed lines on Supplementary figure 4A show the upper limit of quantification, while dashed lines on Supplementary figure 4B show the lower limit of detection, with different colors representing the data of respective subclasses. The IgG2 value of the sample is below the lower limit of detection, therefore it is considered 0.

A

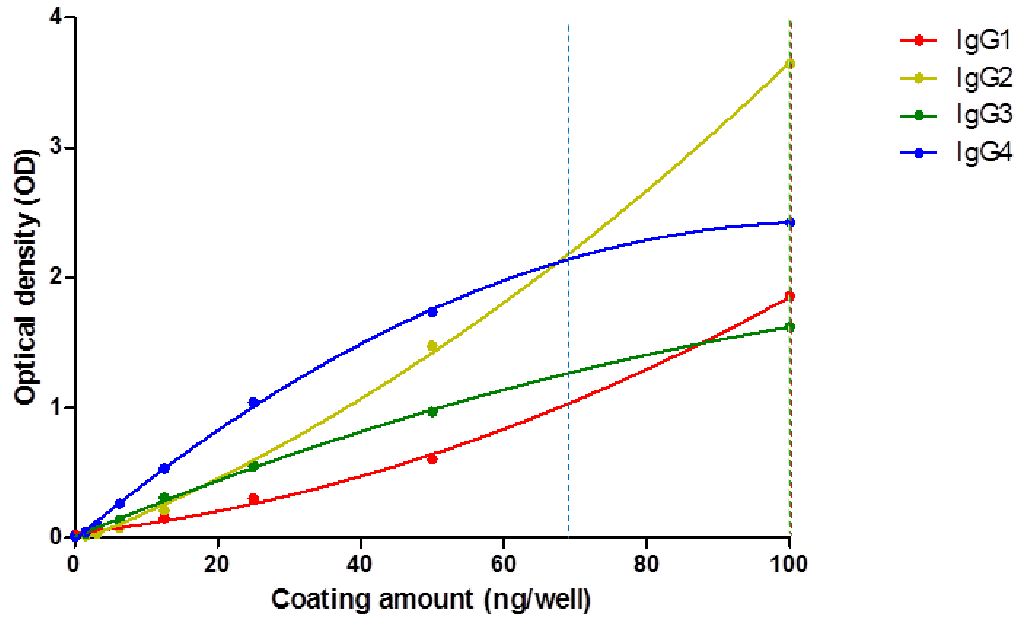

B

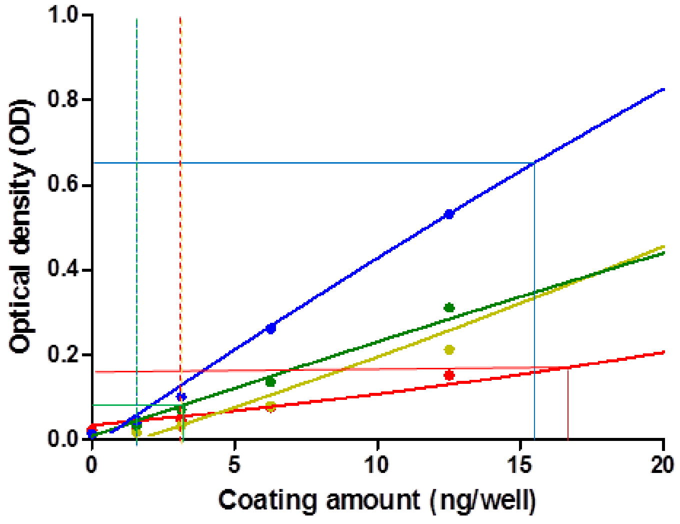

	OD	Amount (ng/well)
IgG1	0.177	17.3
IgG2	0.020	0 (2.5)
IgG3	0.080	3.1
IgG4	0.667	15.6

Supplementary Figure 5. Scheme of calculation of subclass distribution (A) and absolute subclass concentrations (B) of anti-ADAMTS13 IgG autoantibodies. (Results of the patient's sample presented on Supplementary figure 4B are shown.)

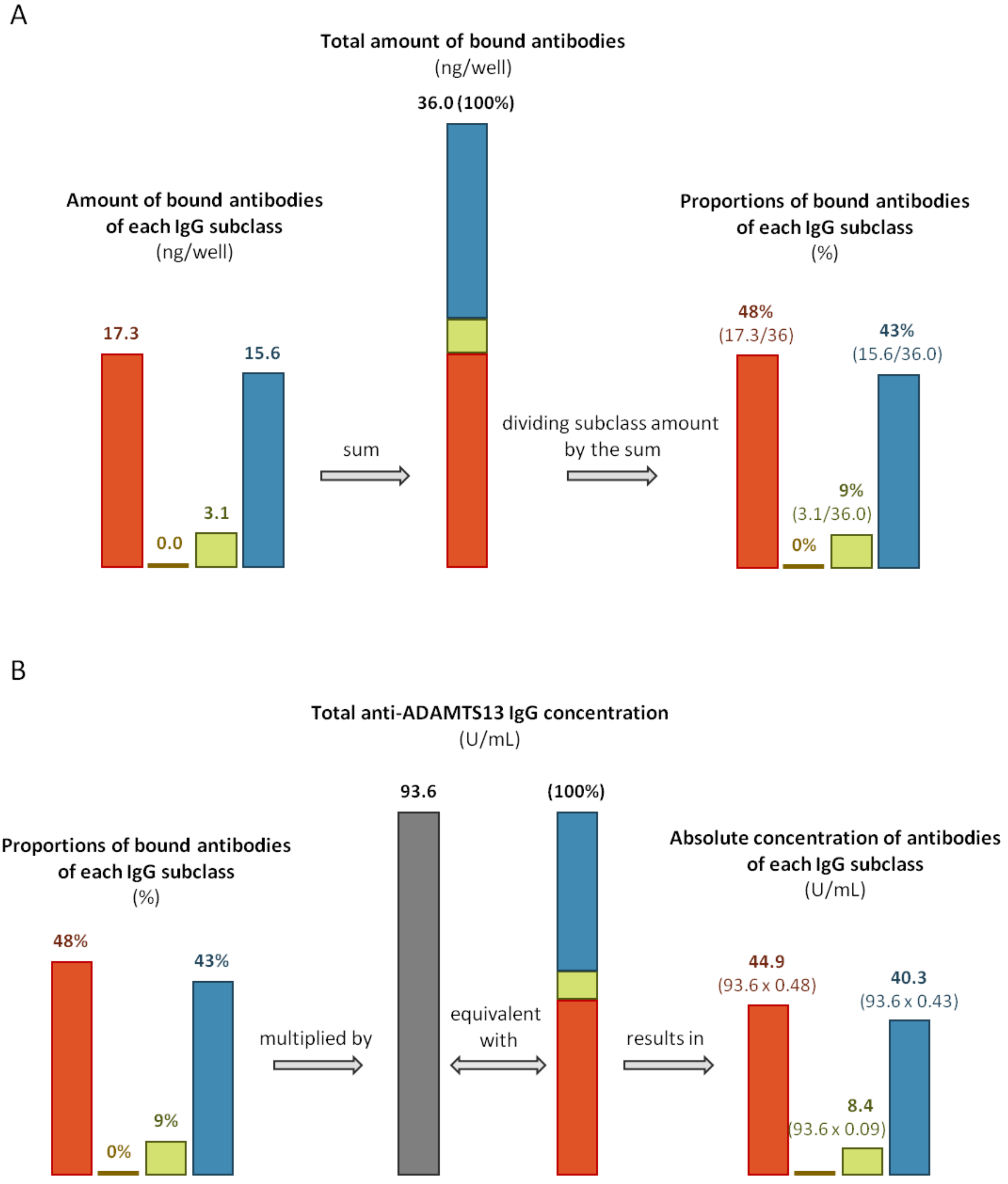

Supplementary Figure 6. Relation of absolute antibody concentrations and amounts of antibodies bound, by subclass. (Points with any value of zero are not shown because of the logarithmic scale.)

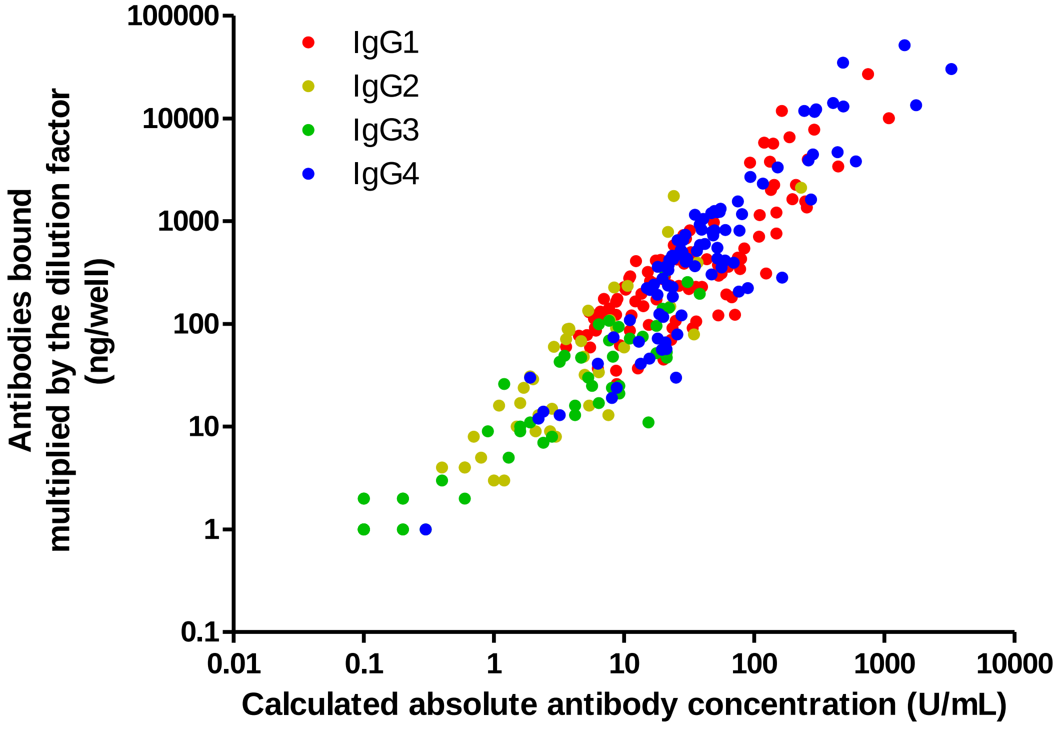

Supplementary Table 1. Proportions of anti-ADAMTS13 IgG subclasses measured in the control sample throughout the third set of measurements. M1-6 marks the distinct measurements. CV% - coefficient of variation expressed as a percentage.

	**M1**	**M2**	**M3**	**M4**	**M5**	**M6**	**Mean**	**CV%**
**IgG1 (%)**	45	44	49	46	48	48	46.7	4.2%
**IgG2 (%)**	0	0	0	0	0	0	0.0	-
**IgG3 (%)**	8	7	8	9	9	6	7.8	14.9%
**IgG4 (%)**	46	49	43	44	43	46	45.2	5.1%

Supplementary Table 2. Intra- and inter-assay coefficients of variation (CV%) of the assay. Intra-assay CV% values were calculated from two sets of triplicate values of three samples. The inter-assay CV% values were calculated from three consecutive measurements of four samples. Intra- and inter-assay coefficients of variation calculated for IgG1, IgG3 and IgG4 subclasses. The concentrations of IgG2 were too low in our samples to adequately calculate the coefficient of variation. The data of samples are listed in order of decreasing average proportions for each subclass.

IgG1	IgG1%	CV% intra	CV% inter
HUN1470	84.4%	1.1%	12.0%
HUN653	57.5%	1.6%	22.5%
HUN86	46.1%	nd	5.8%
HUN1076	42.4%	5.5%	7.9%
**Average**	57.6%	**2.7%**	**12.0%**

IgG3	IgG3%	CV% intra	CV% inter
HUN1076	14.9%	4.6%	5.3%
HUN1470	13.8%	6.3%	6.8%
HUN86	7.7%	nd	7.5%
HUN653	2.5%	25.8%	14.8%
**Average**	9.7%	**12.2%**	**8.6%**

IgG4	IgG4%	CV% intra	CV% inter
HUN86	46.2%	nd	6.5%
HUN1076	42.7%	5.1%	10.5%
HUN653	40.0%	3.6%	13.3%
HUN1470	1.8%	4.9%	49.9%
**Average**	32.7%	**4.5%**	**20.1%**

**Overall**	**-**	**6.5%**	**13.6%**
